# From Bone Marrow Reserve to Metastatic Niche: How Neutrophil-Lineage Cells Shape Skeletal Colonization

**DOI:** 10.3390/ijms27135975

**Published:** 2026-07-03

**Authors:** Fatheia N. Hamza, Mahmoud Zhra, Jasmine Holail, Samaa Alotab, Sidra Alshater, Alaa A. Al-Masud, Khalid Said Mohammad

**Affiliations:** 1 Department of Biochemistry and Molecular Medicine, College of Medicine, Alfaisal University, Riyadh 11533, Saudi Arabia; fahamza@alfaisal.edu (F.N.H.); jholail@alfaisal.edu (J.H.); salotab01@alfaisal.edu (S.A.); 2 Department of Anatomy, College of Medicine, Alfaisal University, Riyadh 11533, Saudi Arabia; mzahra@alfaisal.edu (M.Z.); salshater@alfaisal.edu (S.A.); 3 Tissue Biobank Section, Research Department, Natural and Health Science Research Center, Princess Nourah Bint Abdulrahman University, P.O. Box 84428, Riyadh 11671, Saudi Arabia; aaalmasud@pnu.edu.sa

**Keywords:** bone metastasis, neutrophils, tumor-associated neutrophils (TANs), PMN-MDSCs, NETosis, CXCR2, CXCR4/CXCL12, premetastatic niche, immunotherapy resistance

## Abstract

Bone metastasis develops within a specialized marrow ecosystem where hematopoiesis, immune regulation, vascular trafficking, and skeletal remodeling intersect. Neutrophil-lineage cells occupy a unique position in this setting because they are generated, retained, mobilized, aged, and reprogrammed within the same bone marrow niches that disseminated tumor cells exploit for homing and survival. This review examines how neutrophils, tumor-associated neutrophils, immature neutrophils, low-density neutrophils, and PMN-MDSCs shape skeletal colonization. We discuss tumor-to-marrow signaling, CXCR2-dependent recruitment, CXCR4/CXCL12-mediated marrow retention, neutrophil–circulating tumor cell interactions, vascular arrest, dormancy escape, NET-mediated matrix remodeling, immune suppression, and effects on osteoclast–osteoblast coupling. Evidence is strongest in breast and prostate cancer models, where pathways such as CXCL5/CXCR2, CTNND1–CXCR4/CXCL12, PR3–RAGE, and DKK1–CKAP4–STAT6–CHI3L3 link neutrophil-lineage cells to skeletal progression and immunotherapy resistance. However, several mechanisms, including CTC–neutrophil clustering and NET-driven dormancy awakening, remain partly extrapolated from non-skeletal models. We therefore emphasize evidence hierarchy, methodological limitations, and therapeutic opportunities, arguing that selective reprogramming or functional inhibition of pro-metastatic neutrophil states may be more promising than indiscriminate neutrophil depletion in metastatic bone disease. A clearer understanding of these context-dependent neutrophil programs may help refine biomarker development and guide combination therapies for patients with skeletal metastases.

## 1. Introduction

### 1.1. Bone Metastasis Is Not Just Another Metastatic Site

In this review, skeletal metastasis refers to the dissemination, survival, and growth of malignant cells within bone and bone marrow compartments, including endosteal and perivascular niches. We use the terms “bone metastasis” and “skeletal metastasis” interchangeably to describe metastatic involvement of the mineralized bone, marrow space, and associated stromal, vascular, immune, and bone-remodeling components. Clinically, skeletal metastases may present as osteolytic, osteoblastic, or mixed lesions and are frequently associated with skeletal-related events such as pain, pathological fracture, spinal cord compression, and impaired mobility.

Bone metastasis develops within a highly specialized microenvironment that distinguishes it from metastases in other organs, such as the lung, liver, or brain. The bone marrow is both the primary site of hematopoiesis and a dynamic immune–stromal organ composed of vascular niches, mesenchymal cells, hematopoietic progenitors, and diverse immune populations [1,2]. This ecosystem allows disseminated tumor cells (DTCs) to home, survive, enter dormancy, or progress into clinically detectable metastases [3]. Skeletal colonization is shaped by endosteal and perivascular niches, where tumor cells exploit hematopoietic trafficking mechanisms, including endothelial adhesion molecules and chemokine pathways, to persist within protective marrow compartments [4,5,6].

Clinically, bone metastasis is important because it disrupts skeletal structure and function. Tumor cells disturb the balance between osteoclast-mediated bone resorption and osteoblast-mediated bone formation, generating a “vicious cycle” in which bone destruction releases matrix-stored growth factors that further support tumor survival and progression [7,8,9]. This process contributes to skeletal-related events, including severe bone pain, pathological fractures, spinal cord compression, and functional decline [10]. Bone metastasis, therefore, reflects an interaction between skeletal remodeling and immune regulation, creating conditions that may weaken anti-tumor immunity and promote therapeutic resistance [11,12]. In this context, neutrophils are relevant because they arise from the bone marrow, respond rapidly to tumor-derived inflammatory signals, and can influence metastatic seeding, angiogenesis, immune suppression, skeletal remodeling, and immunotherapy resistance [13,14].

### 1.2. Neutrophils: Abundant, Rapidly Mobilized, and Highly Plastic

Neutrophils are the most abundant circulating leukocytes, accounting for approximately 40–70% of white blood cells. Traditionally, they were regarded as short-lived innate immune effector cells mainly involved in antimicrobial defense [15]. However, growing evidence indicates that neutrophils are highly plastic cells that can adopt distinct functional states in cancer depending on tumor-derived signals and microenvironmental cues [16]. Neutrophils are increasingly recognized as active cancer modulators rather than bystanders [17,18]. This plasticity allows neutrophils to exert either anti-tumor or pro-tumor functions [19]. During early tumor development, neutrophils may contribute to anti-tumor immunity through direct cytotoxicity, reactive oxygen species production, cytokine release, and activation of other immune cells [20]. However, as tumors progress, cancer cells can “educate” neutrophils toward tumor-supportive phenotypes. These tumor-associated neutrophils may promote epithelial–mesenchymal transition, angiogenesis, extracellular matrix remodeling, immune suppression, and metastatic dissemination [21]. Neutrophil behavior is also shaped by tissue-specific signals, including stromal-derived factors, chemokines, and tumor-secreted cytokines, which can reprogram neutrophils toward immunosuppressive or metastasis-promoting states [22].

Tumor-associated neutrophils are often discussed using the simplified N1/N2 framework, where N1-like cells retain cytotoxic or immunostimulatory functions, and N2-like cells support immune suppression, angiogenesis, and therapy resistance [23]. However, this terminology should be used as a conceptual shorthand rather than as a strict lineage classification, because neutrophil states in cancer are better viewed as a continuum shaped by maturation, density, localization, and suppressive function.

One important mechanism through which neutrophils support metastasis is the formation of neutrophil extracellular traps (NETs). NETs can contribute to pre-metastatic niche formation, capture circulating tumor cells (CTCs), and facilitate metastatic outgrowth [13,24].

In bone metastases, neutrophils play a distinctive role at the intersection of hematopoiesis, inflammation, immune suppression, and skeletal remodeling. Their capacity to transition between anti-tumor and pro-metastatic states suggests potential roles across different stages of skeletal colonization, including tumor cell homing, dormancy, osteolysis, immune evasion, metastatic proliferation, and resistance to therapy.

### 1.3. Review Aims and Organization Logic

Unlike most immune cells discussed in bone metastasis, neutrophils are both products of the bone marrow and active remodelers of the metastatic marrow niche. This dual identity places them at a critical interface among emergency granulopoiesis, tumor-cell homing, immunosuppression, NET-driven matrix remodeling, and osteoclast–osteoblast coupling. This review, therefore, focuses on neutrophil-lineage cells as dynamic regulators of skeletal colonization, emphasizing where evidence is bone-specific, where it is extrapolated from other metastatic models, and how these distinctions should guide therapeutic development.

## 2. The Bone Metastatic Ecosystem

### 2.1. Bone Microenvironment Essentials

The bone is not a passive metastatic destination but a dynamic immune, stromal, vascular, and mineralized organ. The bone marrow contains specialized microenvironments that regulate hematopoiesis, immune-cell trafficking, skeletal remodeling, and the survival of disseminated tumor cells (DTCs) [11]. Two major anatomical–functional regions are particularly relevant to bone metastasis: the endosteal niche, located near mineralized bone surfaces, and the perivascular niche, organized around marrow sinusoidal and arteriolar blood vessels. Together, these niches provide adhesion signals, survival cues, dormancy-regulating factors, and access to immune and stromal cell populations that can support metastatic colonization [4,25,26]. Therefore, bone metastasis represents a complex interplay between tumor-cell intrinsic properties and the bone microenvironment rather than tumor characteristics alone.

The endosteal niche contains osteoblast-lineage cells, osteocytes, osteoclasts, bone-lining cells, and the mineralized bone matrix [27]. Osteoblast-lineage cells regulate bone formation and contribute to hematopoietic and metastatic niches through extracellular matrix production, osteogenic signaling, and regulation of the RANKL/osteoprotegerin (OPG) balance, which governs osteoclastogenesis [28,29]. Osteoclasts mediate bone resorption and are central to osteolytic bone destruction [30]. The mineralized bone matrix serves as a reservoir for bioactive factors, particularly TGF-β, which are released during osteoclast-mediated bone resorption and create a feedback loop that influences both bone remodeling and tumor growth [31].

The perivascular niche comprises sinusoidal and arteriolar endothelial cells, pericytes, mesenchymal stromal cells (including leptin receptor-positive (LepR^+^) cells and CXCL12-abundant reticular (CAR) cells), macrophages, hematopoietic stem and progenitor cells, and developing myeloid cells [32,33]. This compartment is important because it regulates marrow trafficking, hematopoietic-cell retention, vascular access, and chemokine gradients used by both normal and malignant cells [12,34]. Furthermore, CXCL12/CXCR4 signaling is particularly significant, as it is involved in the homing and retention mechanisms of hematopoietic cells, which are also exploited by metastatic tumor cells [35]. Moreover, LepR^+^ perivascular stromal cells, which include CXCL12-abundant reticular (CAR) cells, are primary sources of niche factors such as CXCL12 and SCF, sustaining a chemokine-rich microenvironment in which CXCL12/CXCR4 signaling can anchor disseminated tumor cells (DTCs) and influence their persistence or dormancy [36,37].

Other stromal components, including marrow adipocytes, may influence inflammatory tone and tumor metabolism, but direct adipocyte–neutrophil mechanisms in skeletal metastasis remain insufficiently defined [38,39].

The classical vicious cycle is best seen in osteolytic bone metastasis. Tumor-derived factors, especially PTHrP, together with IL-6 and IL-11, increase the RANKL/OPG ratio in osteoblast-lineage cells, driving osteoclast formation and activation [40]. These activated osteoclasts then resorb bone and release matrix-stored growth factors, particularly TGF-β, which feed back to stimulate tumor-cell growth and further osteolytic signaling. This self-reinforcing loop links tumor expansion with bone destruction and reflects a broader osteo-immune circuit involving tumor, immune, and bone-remodeling cells [41].

**Figure 1:**
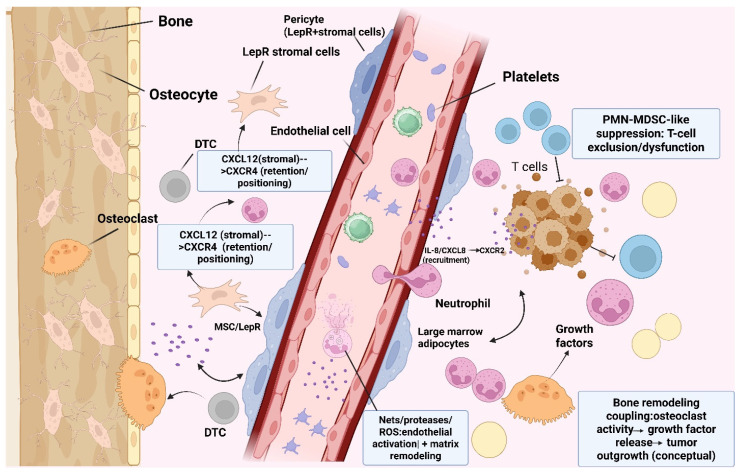
Neutrophil-lineage cells within the metastatic bone marrow niche. Schematic representation of the bone metastatic microenvironment as an immune, vascular, stromal, and skeletal-remodeling niche. Disseminated tumor cells (DTCs) localize near endosteal and perivascular compartments, where leptin receptor-positive (LepR+) stromal cells, pericytes, endothelial cells, osteocytes, osteoclasts, and marrow adipocytes contribute to niche organization. Stromal CXCL12 can engage CXCR4 to support retention and positioning of both tumor cells and neutrophil-lineage cells within marrow compartments. Tumor- and niche-derived inflammatory signals, including IL-8/CXCL8–CXCR2-associated recruitment cues, promote neutrophil accumulation and activation. Once positioned in the niche, neutrophils may contribute through NET formation, protease release, ROS production, endothelial activation, and matrix remodeling. In established disease, immature neutrophils or PMN-MDSC-like states may promote T-cell exclusion or dysfunction, thereby supporting immune evasion. Neutrophils may also modify, rather than replace, the classical tumor–osteoblast–osteoclast vicious cycle by amplifying inflammatory and proteolytic signals that influence osteoclast activity, growth-factor release, and tumor outgrowth. Abbreviations: DTC, disseminated tumor cell; LepR, leptin receptor; NET, neutrophil extracellular trap; ROS, reactive oxygen species; PMN-MDSC, polymorphonuclear myeloid-derived suppressor cell. Created in BioRender. Mohammad, K. (2026) https://BioRender.com/r6d2y4y (accessed on 29 June 2026).

Within this circuit, immune cells in the bone marrow act as important modifiers of the core tumor–osteoblast–osteoclast axis. Macrophages, T-cell subsets, myeloid-derived suppressor cells, and neutrophils collectively shape inflammatory signaling, osteoclastogenesis, endothelial behavior, and stromal activation through cytokines, chemokines, proteases, and angiogenic factors [11,41]. Notably, among these populations, neutrophils additionally contribute through NET-related programs, which represent a distinct effector module emphasized throughout this review [42]. Neutrophils may influence both osteolytic and osteoblastic bone lesions, although the strength of evidence differs by tumor type and metastatic context [43,44]. These immune inputs are increasingly recognized as biologically important; however, the canonical core of the cycle remains the tumor cell–osteoblast–osteoclast–TGF-β axis [45]. These niche-level interactions are summarized schematically in Figure 1.

### 2.2. Osteolytic Versus Osteoblastic Lesions: Where Neutrophils May Diverge

Bone metastases are classified as osteolytic, osteoblastic, or mixed lesions based on the balance between bone destruction and formation [46]. Osteolytic lesions, characterized by increased osteoclast-mediated bone resorption, are commonly associated with breast, lung, and renal cancers [47,48], while osteoblastic lesions result from enhanced osteoblast activity and are typically seen in prostate cancer [49]. Mixed lesions combine both features [50]. These categories, however, are not absolute: osteoblastic lesions still involve active osteoclast-mediated resorption, and osteolytic lesions can include abnormal osteoblast responses, since bone formation and resorption remain biologically coupled even in pathological remodeling [51,52].

The distinction between osteolytic and osteoblastic lesions has important implications for neutrophil function, since neutrophils may engage different remodeling programs depending on the dominant lesion phenotype [53]. In osteolytic lesions, neutrophils may contribute through inflammatory amplification, endothelial activation, extracellular matrix remodeling, and indirect support of osteoclastogenesis [53,54]. Neutrophil-associated cytokines, chemokines, reactive oxygen species, and proteases may enhance stromal inflammation and reinforce signals that favor osteoclast differentiation and activity [55]. These mechanisms are introduced here only conceptually; detailed discussion of CXCR2 ligands, NETosis, granule proteases, and PMN-MDSC–like immunosuppressive functions is reserved for later mechanistic sections.

In osteoblastic lesions, particularly those arising from prostate cancer, neutrophil functions are likely to diverge from a purely osteoclast-supportive role. These lesions are shaped by abnormal osteoblast activation, dysregulated osteogenic signaling, and persistent coupling between bone formation and resorption [56]. In this context, neutrophils may contribute not only through inflammatory support of osteoclast activity but also through effects on tumor-cell motility, vascular remodeling, local immunosuppression, and modulation of osteoblast-lineage cells [57]. This framework is introduced here only conceptually and is developed further in the tumor-type section. However, direct head-to-head studies comparing neutrophil function in osteolytic versus osteoblastic skeletal lesions remain limited, and this distinction should be treated as a working framework rather than a settled mechanism.

## 3. Neutrophil Biology

### 3.1. Developmental Continuum: From Marrow Progenitors to Mature and Aged Neutrophils

#### 3.1.1. Granulopoiesis and Bone Marrow Differentiation

Neutrophils are continuously generated within the bone marrow niche through granulopoiesis, a tightly regulated developmental process controlled by granulocyte colony-stimulating factor (G-CSF) and lineage-specific transcriptional programs [58]. Hematopoietic stem and progenitor cells differentiate through common myeloid progenitors and granulocyte-monocyte progenitors into progressively mature neutrophil populations [58,59].

Refined models of neutrophil development identify discrete stages, including proliferative neutrophil precursors (preNeu), immature post-mitotic neutrophils, and terminally mature neutrophils [60]. Maturation is accompanied by the acquisition of migratory and effector functions, as well as transcriptional and metabolic reprogramming rather than purely morphological changes [60,61].

#### 3.1.2. Mobilization and Retention: The CXCR4–CXCL12 Axis

The CXCR4 governs neutrophil retention and release from the bone marrow–CXCL12 chemokine axis, which plays a central role in maintaining marrow homeostasis and is also highly relevant to bone metastasis. CXCL12, abundantly produced by bone marrow stromal cells, engages CXCR4 to retain neutrophils within the niche, whereas egress into the circulation is driven by CXCR2 signaling in response to ligands such as CXCL1 and CXCL2 [58]. This balance ensures controlled neutrophil trafficking under homeostatic conditions. This regulatory balance is frequently disrupted by cancer. Tumor-derived factors, including G-CSF, enhance neutrophil mobilization by lowering the threshold for marrow release and amplifying mobilizing signals, thereby increasing the circulation of immature neutrophils [58,60]. Notably, the same CXCR4–CXCL12 axis is exploited by tumor cells to home to and colonize the bone marrow, suggesting that neutrophil trafficking and tumor dissemination are governed by overlapping mechanisms. This convergence positions the bone marrow as a dynamic interface where altered neutrophil output may contribute to metastatic niche conditioning [62].

#### 3.1.3. Circadian Regulation and Neutrophil Aging

Neutrophil production and release follow circadian rhythms, partly driven by oscillations in CXCL12 and CXCR2 signaling. After entering circulation, neutrophils undergo time-dependent “aging,” characterized by increased CXCR4 expression and reduced CD62L levels, thereby facilitating their return to the bone marrow and clearance [63,64]. In cancer, disruption of circadian regulation may alter neutrophil trafficking and functional states, thereby influencing tumor progression and metastatic spread [65]. Given that neutrophils return to the bone marrow during aging, this process may also contribute to remodeling of the marrow microenvironment and impact interactions with disseminated tumor cells [66].

### 3.2. Neutrophil States and Subsets in Cancer

Neutrophils in cancer comprise a heterogeneous spectrum of cellular states shaped by developmental stage and tumor-derived signals [67]. Key populations include tumor-associated neutrophils (TANs), polymorphonuclear myeloid-derived suppressor cells (PMN-MDSCs), low-density neutrophils (LDNs), and immature neutrophils [67]. These are best viewed as a continuum rather than discrete subsets, particularly in advanced disease and metastatic settings [68]. Importantly, these terms reflect overlapping anatomic, functional, density-based, and developmental designations rather than entirely distinct populations: TANs primarily represent an anatomic designation, PMN-MDSCs a functional state, LDNs a density-based fraction, and immature neutrophils a developmental stage [13,67]. Consequently, functional and transcriptional profiling are often required for accurate characterization of neutrophil states within the tumor and metastatic microenvironment [68].

Tumor-associated neutrophils (TANs) are a major component of the tumor microenvironment (TME) and display functional plasticity [67]. Although they can exert anti-tumor effects, TANs in established tumors more commonly promote angiogenesis, matrix remodeling, and immunosuppression [69]. Single-cell RNA sequencing studies have identified distinct TAN clusters enriched for hypoxia signaling, metabolic rewiring, and pro-angiogenic programs, features highly relevant to metastatic growth within hypoxic niches such as bone [67,70].

Polymorphonuclear myeloid-derived suppressor cells (PMN-MDSCs) are defined functionally by their ability to suppress T-cell responses [71]. They share phenotypic markers with conventional neutrophils (e.g., CD11b^+^CD15^+^/CD66b^+^), limiting clear discrimination [72,73]. Transcriptomic analyses show that circulating PMN-MDSCs and intratumoral TANs share programs associated with hypoxia, angiogenesis, and metabolic adaptation, supporting a close relationship between these populations [71]. Functionally, PMN-MDSCs promote immune evasion, tumor progression, and metastatic dissemination, including through pathways such as C-X-C motif chemokine ligand 2–C-X-C motif chemokine receptor 2 (CXCL2–CXCR2) signaling, which are relevant to tumor–bone interactions [72]. In human studies, PMN-MDSCs are commonly described as CD11b+CD14−CD15+ or CD11b+CD14−CD66b+ cells, but functional evidence of immunosuppression remains necessary, as these markers overlap with those of conventional neutrophils.

Low-density neutrophils (LDNs) represent a heterogeneous mixture of immature and mature neutrophils, including PMN-MDSCs, and expand in cancer due to dysregulated myelopoiesis [71]. Importantly, suppressive neutrophils are not confined to this fraction, highlighting limitations of density-based classification [13]. Tumor-driven granulopoiesis also increases circulating immature neutrophils with pro-tumorigenic properties, which may contribute to pre-metastatic niche formation in the bone marrow [13,68].

A major limitation in the field is the reliance on phenotypes to define neutrophil subsets. Common markers lack specificity, and proposed candidates, such as lectin-type oxidized low-density lipoprotein receptor 1 (LOX-1), are not consistently applicable across tumor types [71]. Consequently, immunosuppressive function remains the defining feature of PMN-MDSCs, underscoring the lack of standardized nomenclature.

### 3.3. Neutrophil Effector Modules: A Mechanistic Toolkit

Neutrophils deploy multiple effector mechanisms that extend beyond antimicrobial defense to regulate tumor progression, metastatic seeding, and microenvironmental remodeling [68]. These functions are highly context-dependent and often converge to influence tumor cell behavior, extracellular matrix (ECM) dynamics, and immune responses [16].

#### 3.3.1. NETosis and Neutrophil Extracellular Traps (NETs)

NETosis results in the release of chromatin structures decorated with granular proteins, including neutrophil elastase (NE), myeloperoxidase (MPO), histones, and cathepsin G [74]. NETs can entrap circulating tumor cells and facilitate metastatic seeding by promoting tumor cell adhesion and extravasation [75]. NET-associated proteases additionally contribute to extracellular matrix remodeling and establishment of a pro-thrombotic microenvironment that supports metastatic dissemination [75].

#### 3.3.2. Degranulation and Proteases

Neutrophil degranulation releases proteases such as neutrophil elastase (NE), cathepsin G, proteinase 3 (PR3), and matrix metalloproteinase-9 (MMP-9), which promote tumor progression through extracellular matrix (ECM) degradation, invasion, angiogenesis, and epithelial–mesenchymal transition (EMT). NE and cathepsin G contribute to matrix remodeling and modulation of tumor cell adhesion and migration. At the same time, MMP-9 facilitates angiogenesis and tissue remodeling by degrading ECM components and releasing matrix-bound growth factors. In addition, these proteases regulate cytokine activity and inflammatory microenvironmental signaling [76]. PR3 becomes particularly relevant in prostate cancer bone metastasis, where PR3–RAGE signaling promotes tumor-cell motility and homing toward the bone marrow microenvironment.

#### 3.3.3. ROS/RNS and Signaling Effects

Neutrophil-derived reactive oxygen and nitrogen species (ROS/RNS) function not only as antimicrobial mediators but also as potent regulators of tumor progression and stromal remodeling. Sustained ROS production induces oxidative DNA damage and genomic instability within tumor cells, thereby promoting mutagenesis and oncogenic signaling pathways [16]. ROS additionally suppresses anti-tumor immunity by impairing T-cell receptor signaling, reducing T-cell proliferation, and promoting T-cell exhaustion within the tumor microenvironment [77]. ROS signaling also promotes NET formation, linking oxidative stress to metastasis and tumor-associated thrombosis [74]. In skeletal metastasis, these ROS/RNS-dependent mechanisms are plausible contributors to immune evasion, stromal remodeling, and osteoclast-associated signaling, although direct bone-specific validation remains incomplete.

#### 3.3.4. Cytokines and Chemokines

Neutrophils produce and respond to a diverse network of cytokines and chemokines that regulate myeloid recruitment, inflammation, and metastatic progression. Chemokines, including IL-8 (CXCL8), CXCL1, CXCL2, and CXCL5, promote neutrophil trafficking through CXCR1/2 signaling and establish feed-forward inflammatory loops within the tumor microenvironment [64]. Tumor-derived granulocyte colony-stimulating factor (G-CSF), granulocyte-macrophage colony-stimulating factor (GM-CSF), and interleukin-1β (IL-1β) further amplify emergency granulopoiesis and mobilization of immature neutrophils from the bone marrow [78,79]. Damage-associated inflammatory mediators such as S100A8/A9 also promote the recruitment and activation of suppressive myeloid populations while enhancing pre-metastatic niche formation within bone [78]. These inflammatory circuits link systemic cancer-associated inflammation with local remodeling of the metastatic microenvironment and sustained myeloid cell infiltration.

#### 3.3.5. Immune Suppression

Neutrophils suppress anti-tumor immunity through multiple complementary mechanisms, including arginase-1 (ARG1)- mediated immunosuppression, ROS production, immune checkpoint signaling, and physical exclusion of cytotoxic immune cells. ARG1 depletes extracellular arginine, impairing T-cell proliferation and effector function, whereas ROS disrupts T-cell receptor signaling and promotes T-cell dysfunction and exhaustion [77]. Tumor-associated neutrophils and PMN-MDSCs also express programmed death ligand 1 (PD-L1), thereby directly suppressing cytotoxic T-cell activity through checkpoint signaling pathways. Neutrophils further contribute to immunosuppressive tumor microenvironments through inflammatory cytokine production and extracellular matrix remodeling, thereby limiting effective immune cell infiltration [80]. Collectively, these suppressive functions facilitate immune evasion and support metastatic progression, particularly within chronically inflamed metastatic environments such as bone.

## 4. Recruitment to Bone and Education by Tumor

### 4.1. Systemic Mobilization: Tumor-to-Marrow Signaling

Primary tumors can systemically reprogram neutrophil production and release from the bone marrow through a tumor-to-marrow signaling axis that precedes overt metastatic colonization [81,82]. Under homeostatic conditions, neutrophil retention in the bone marrow is largely governed by the CXCR4-CXCL12 axis, whereas tumor-derived inflammatory mediators, such as G-CSF, disrupt this balance, suppress marrow retention programs, and enhance granulopoiesis and peripheral mobilization [83,84]. Accumulating evidence indicates that tumors can alter neutrophil output at early stages of differentiation, generating heterogeneous populations of immature and pathologically activated neutrophils with enhanced survival, trafficking capacity, and pro-metastatic function [81].

### 4.2. Homing/Trafficking Axes That Matter in Metastasis

Chemokine networks are central to neutrophil trafficking to both the bone marrow and metastatic niches. The CXCR4-CXCL12 axis is especially important because it regulates hematopoietic stem and progenitor cells (HSPCs) retention, neutrophil retention in the marrow, and their release into circulation, while the CXCL1/CXCL2-CXCR2 pathway further supports neutrophil mobilization [85,86].

**Figure 2:**
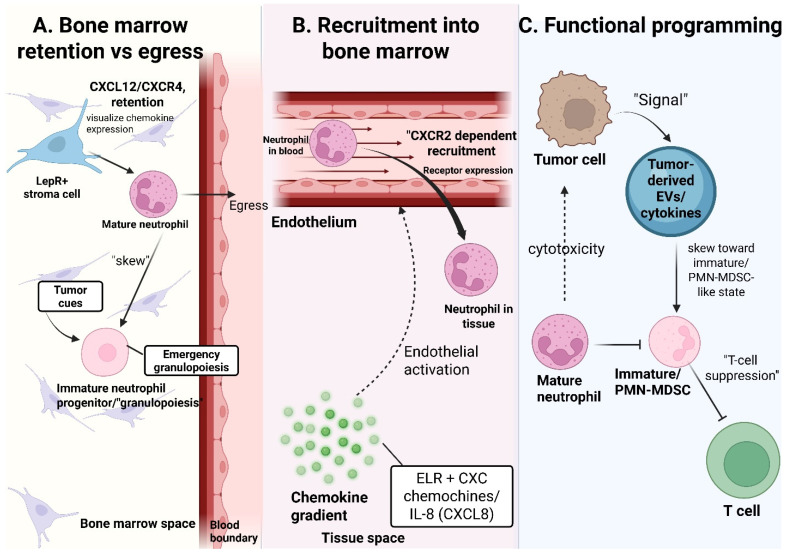
Bone marrow retention, recruitment, and functional programming of neutrophil-lineage cells. This schematic summarizes three linked processes through which neutrophil-lineage cells may influence skeletal metastasis. (**A**) Under homeostatic conditions, CXCL12 produced by LepR+ stromal cells and other marrow stromal populations engages CXCR4 to retain mature neutrophils within the bone marrow, whereas tumor-derived cues can promote emergency granulopoiesis and expansion of immature neutrophil-lineage cells. (**B**) During metastatic progression, inflammatory chemokine gradients, including ELR+ CXC chemokines such as IL-8/CXCL8, promote CXCR2-dependent recruitment of neutrophils across activated endothelium into marrow or metastatic tissue compartments. (**C**) Tumor-derived extracellular vesicles, cytokines, and other soluble mediators can functionally skew neutrophils away from cytotoxic or immune-supportive activity and toward immature or PMN-MDSC-like suppressive states. These programmed neutrophil-lineage cells can suppress T-cell responses and contribute to immune evasion within the metastatic bone microenvironment. Abbreviations: LepR, leptin receptor; CXCL12, C-X-C motif chemokine ligand 12; CXCR4, C-X-C chemokine receptor 4; CXCR2, C-X-C chemokine receptor 2; IL-8/CXCL8, interleukin-8/C-X-C motif chemokine ligand 8; ELR+ CXC chemokines, CXC chemokines containing the Glu-Leu-Arg motif; PMN-MDSC, polymorphonuclear myeloid-derived suppressor cell. Created in BioRender. Mohammad, K. (2026) https://BioRender.com/7wa4q5v (accessed on 29 June 2026).

CXCR4/CXCL12 signaling axis is a major mediator of metastasis, particularly in breast and prostate cancer, homing to the bone. CXCR4 is markedly upregulated across more than 20 human tumors, including breast and prostate cancers. The highly metastatic MDA-MB-231 cells expressed the highest levels of CXCR4 mRNA, which correlated with a strong enrichment of metastasis-related signaling pathways [87,88]. Besides their role in the CXCR4/CXCR12 axis in neutrophil recruitment, they also play a role in the recruitment of osteoclast precursors and osteoprogenitors, and in the coupling of bone resorption and formation [89]. In the zebrafish model, cxcr4b, the ortholog of human CXCR4, is highly expressed in neutrophils, and loss of cxcr4b impairs experimental micrometastasis formation, indicating that this pathway is essential for metastasis and neutrophil motility [85]. Targeting the CXCR4 receptor in the castration-resistant prostate cancer (CRPC) model attenuates metastasis development [88]. The major axes of retention, recruitment, and functional programming are summarized in Figure 2.

### 4.3. Pre-Metastatic Niche Formation

The premetastatic niche is a specialized microenvironment that forms in distant organs before tumor cell arrival, characterized by immunosuppression, increased vascular permeability, angiogenesis, stromal activation, extracellular matrix remodeling, and recruitment of immune cells.

Neutrophils play an important role in this process by migrating to premetastatic sites in response to chemotactic signals [90,91]. G-CSF promotes neutrophil mobilization and supports their recruitment to distant organs even before tumor cells reach those sites. In addition, neutrophils accumulate in large numbers within pre-metastatic organs, supporting evidence that they contribute to pre-metastatic niche formation [86,91].

To remodel the local microenvironment and support angiogenesis, they secrete CXCL8 and multiple proteases, including MMP-9, which degrade extracellular matrix and liberate angiogenic factors such as VEGF and FGF2, thereby promoting vascularization at metastatic sites [90,91]. Activated endothelial cells recruit neutrophils by increasing adhesion molecules such as VCAM-1 and ICAM-1 and releasing neutrophil-attracting chemokines. Recruited neutrophils then adhere to ICAM-1 and degranulate, releasing elastase and other proteases that weaken vascular and lymphatic barriers. This increases permeability and helps tumor cells cross the endothelium, enter distant organs, and promote metastasis [86]. NETs further reshape the niche, facilitating tumor cell extravasation [91]. They provide an adhesive scaffold of DNA and associated proteins that supports tumor cell attachment to the vessel wall and promotes metastatic seeding [92].

Additional tumor-derived soluble factors, including G-CSF, CCL2, and TIMP-1, may recruit or condition neutrophil-lineage cells within pre-metastatic or early metastatic niches. Once recruited, neutrophils can express or induce the expression of metastasis-associated mediators such as Bv8, MMP-9, S100A8, and S100A9, thereby supporting extracellular matrix remodeling, angiogenesis, and local immunosuppression. However, the degree to which these programs operate specifically in bone remains incompletely defined [93].

Immature neutrophils become predominant as bone metastases progress, contributing to tumor growth and immunosuppression. In *an* in vivo model, Bone lesion neutrophils exhibit stronger immunosuppressive functions than those from primary tumors or lung metastases. Immature neutrophils promote immune suppression in bone metastases via CHI3L3, and DKK1 blockade promotes neutrophil maturation, enhances anti-tumor immunity, and improves responses to immune checkpoint blockade, offering promising therapeutic strategies [94].

## 5. Neutrophils Across the Metastatic Cascade in Bone

In cancer, TANs are recruited and reprogrammed by tumor- and niche-derived signals, acquiring context-dependent antitumor or protumor phenotypes that can influence tumor initiation, proliferation, recurrence, invasion, metastasis, angiogenesis, immune evasion, and tissue remodeling [23]. This functional plasticity is commonly conceptualized along an N1/N2-like spectrum, in which N1-like neutrophils retain cytotoxic and immunostimulatory properties, whereas N2-like neutrophils, often promoted by factors such as TGF-β, promote immunosuppression, angiogenesis, cancer cell motility, EMT, and metastatic progression [14,95,96]. Among neutrophil effector programs, NETosis has emerged as a central mechanism linking inflammation to metastasis. During NETosis, activated neutrophils externalize decondensed chromatin decorated with granule-derived proteins, including neutrophil elastase, myeloperoxidase, cathepsin G, lactoferrin, and gelatinase, generating extracellular DNA-protein scaffolds originally described for microbial trapping but now implicated in tumor-cell adhesion, vascular arrest, immune evasion, dormancy escape, and metastatic niche formation [97]. NETosis may occur through suicidal, vital, or mitochondrial programs: suicidal NETosis is a slower lytic process involving nuclear chromatin release and neutrophil death, vital NETosis allows rapid DNA release with relative preservation of neutrophil viability, and mitochondrial NETosis releases mitochondrial rather than nuclear DNA [98]. Building on this plasticity, the following sections integrate available evidence from bone-specific and non-skeletal metastatic models to examine how neutrophils may shape the metastatic cascade toward bone, from primary-site priming and CTC survival to marrow vascular arrest, extravasation, colonization, dormancy escape, and skeletal niche remodeling, while clearly acknowledging where bone-specific evidence remains limited and where mechanistic hypotheses must be inferred from broader neutrophil biology.

### 5.1. Local Invasion and Intravasation

Although neutrophil priming at the primary tumor is best characterized in non-skeletal models, selected tumor contexts already point to mechanisms that converge on bone tropism, with TNBC as one example. Studies of TNBC suggest a more direct link to bone metastasis, identifying CTNND1 downregulation in patients with bone-only metastasis and associating it with higher rates of bone metastasis, shorter OS, and shorter DMFS. Mechanistically, CTNND1 loss promotes EMT and bone homing by activating the PI3K/AKT/HIF-1α/CXCR4 pathway. After tumor cells reach bone, CTNND1-low cells secrete higher levels of GM-CSF and IL-8, thereby recruiting immature myeloid cells, particularly neutrophils, and impairing cytotoxic T-cell proliferation and function [99,100]. This example provides a useful bridge between primary-site tumor-cell programming and the bone metastatic niche. However, it does not imply that all neutrophil-driven EMT programs are bone-specific; rather, it suggests that in selected tumor contexts, EMT, CXCR4-associated bone homing, and neutrophil recruitment may converge during skeletal dissemination.

### 5.2. Survival in Circulation and CTC–Neutrophil Interactions

Once in circulation, neutrophils may support CTC survival by forming heterotypic clusters that protect tumor cells from shear stress, anoikis, and immune elimination; such clusters show greater metastatic potential than single CTCs owing to increased resistance to apoptosis and immune clearance, stronger endothelial adhesion, and enhanced extravasation and colonization capacity [101]. Among non-tumor cells, neutrophils appear to be one of the most frequent CTC-associated populations; scRNA sequencing of CTC-WBC clusters in breast cancer patients revealed that most form with neutrophils, and their presence correlated with worse OS and transcriptional enrichment for cell-cycle progression [102]. Mechanistically, CTC–neutrophil clustering is supported by coordinated adhesion and cytokine signaling. Neutrophils within CTC clusters can express TNF-α, OSM, IL-1, and IL-6, while matched tumor cells express the corresponding receptors.

TANs can also impair NK-cell cytotoxicity and infiltration, downregulate CCR1, and reduce responsiveness of NK-activating receptors, including NKp46 and NKG2D [103]. In parallel, CTCs expressing CD47 can engage SIRPα on neutrophils to suppress phagocytosis, while CD44v6 or MUC1 on CTCs can bind neutrophil selectins to promote clustering and metastatic efficiency [104].

Although most evidence for CTC-neutrophil clustering and immune shielding comes from non-skeletal metastatic settings, these mechanisms may still be relevant before skeletal colonization, as tumor cells must first survive in circulation, evade immune clearance, and establish stable endothelial interactions before reaching any distant niche.

### 5.3. Bone Marrow Vascular Arrest and Extravasation

The bone marrow vasculature offers a unique site for metastasis, but the role of neutrophils in tumor-cell arrest and extravasation here remains poorly understood. Unlike many non-skeletal vessels, the metaphyseal vasculature of long bones naturally expresses adhesion molecules such as P-selectin, E-selectin, ICAM, and VCAM, which are usually induced only during inflammation in other organs [105]. The fenestrated marrow vessels and abundant sinusoids may foster an environment conducive to CTC arrest, endothelial interaction, and entry into the bone marrow [105,106]. While neutrophil-assisted vascular arrest is better studied outside bone, the shared adhesion molecules involved in CTC-neutrophil clustering, especially VCAM-1/ICAM-1 and integrins, which are constitutively expressed in the metaphyseal marrow vasculature, suggest that neutrophil-CTC adhesive programs could facilitate tumor cell anchoring within marrow vessels.

Extravasation generally requires tumor-cell margination, endothelial adhesion, vascular arrest, endothelial barrier remodeling, and transendothelial migration into the surrounding tissue [107,108]. Neutrophils may contribute to this process through their own adhesion machinery: L-selectin can mediate rolling, while LFA-1, Mac-1, β2-integrins, and CD18 can interact with endothelial ICAM-1 or tumor-cell adhesion molecules, helping CTC clusters anchor to the endothelium, resist shear stress, and extravasate [109,110]. Tumor-derived IL-8 can increase Mac-1 expression on neutrophils, strengthening their binding to ICAM-1 on endothelial cells [107]. Neutrophils may also promote CTC adhesion through interactions between tumor-cell sialyl-Lewis x-containing selectin ligands and endothelial E-selectin, further supporting vascular attachment and transmigration [107]. However, whether these events occur preferentially or uniquely within marrow sinusoids remains to be directly established.

Neutrophil effector mechanisms active at primary sites are redeployed during distant extravasation, where IL-8, MMP8, and MMP9 increase vascular permeability and facilitate CTC transmigration [110]; tumor-conditioned neutrophils further support this process through delayed apoptosis and upregulated adhesion molecules, and once arrested within tumor-cell clusters, actively migrate via IL-8 and CXCL1 signaling to disrupt the endothelial barrier and enhance extravasation locally [107,109].

Evidence from bone-relevant systems suggests that neutrophil-vascular interactions in the skeletal niche may be more complex than a purely prometastatic model. In a vascularized breast cancer-to-bone microfluidic model, metastatic cells disrupted vascular architecture and enhanced neutrophil extravasation. Importantly, neutrophil perfusion increased vascular permeability and also promoted N1-like cytotoxicity against cancer cells through TNF/FAS-associated pathways [111]. This finding is important because it shows that in bone metastasis, neutrophil-induced vascular permeability can coexist with antitumor activity.

A skeletal-directed neutrophil-dependent mechanism involves proteinase 3, encoded by PRTN3 and stored in neutrophil azurophil granules. Proteinase 3 can interact with tumor-cell RAGE to promote prostate cancer migration toward the bone microenvironment and induce bone metastasis by activating the p44/42 and JNK1 pathways [99]. This is particularly relevant because RAGE can bind multiple inflammatory and neutrophil-associated ligands, including HMGB1, S100 proteins, cathepsin G, neutrophil elastase, and proteinase 3 [112], suggesting that RAGE may represent a shared interface through which tumor cells, neutrophil granule proteins, inflammatory ligands, and the bone metastatic niche communicate.

Finally, tumor-cell homing to bone is also influenced by marrow-specific chemokine biology. CXCL12 is expressed in specific areas of the bone marrow and recruits CXCR4-expressing tumor cells, thereby supporting their osteotropic tendency and interactions with the marrow microenvironment [105]. Although this axis is not exclusive to neutrophils, it is important for this review because CXCR4/CXCL12 also regulates neutrophil marrow retention and trafficking, as explained above. Thus, tumor-cell homing, neutrophil positioning, and vascular entry into bone may converge through overlapping chemokine and adhesion networks.

### 5.4. Colonization: Dormancy Versus Outgrowth in the Bone Niche

#### 5.4.1. Neutrophil-Mediated Immune Surveillance and Dormancy Maintenance

At metastatic sites, neutrophil function is not uniform; in some contexts, they retain cytotoxic and immune-surveillance roles, whereas in others they adopt suppressive or trophic programs that favor DTC survival [86]. This duality is evident in G-CSF-driven neutrophilia, where neutrophils are antimetastatic in NK-cell-deficient mice but prometastatic in NK-competent mice. They suppress the more potent tumoricidal activity of NK cells despite retaining direct cytotoxicity, with ROS signaling underlying both roles [113]. In prostate cancer bone metastases, bone-derived neutrophils initially exhibit antitumor cytotoxicity, inducing PCa apoptosis via STAT5 targeting. Their early depletion accelerates bone tumor growth; however, as tumor burden increases, these TANs lose cytotoxic potential and acquire immature, G-MDSC-like features with diminished activation and NET formation [95]. Therefore, neutrophil effects on bone colonization must be interpreted relative to disease stage, maturation state, and the broader immune composition of the marrow niche.

Neutrophils may also influence whether DTCs remain dormant or re-enter the proliferative state. Bone dormancy is regulated by osteoblast-derived signals, including Wnt5a, CXCL12-CXCR4, and TGFβ-p38MAPK-pRB pathways, as well as by transcriptional programs such as NR2F1. NR2F1-positive bone marrow DTCs are associated with reduced metastatic progression in breast cancer [114,115,116,117,118]. These pathways define the skeletal dormancy landscape into which neutrophil-derived inflammatory and proteolytic signals may be introduced.

#### 5.4.2. Neutrophil-Mediated Awakening and Metastatic Outgrowth

As metastasis progresses, neutrophils increasingly acquire immature and suppressive phenotypes that favor tumor outgrowth. In breast cancer bone metastasis, DKK1 drives neutrophil immaturity via the DKK1-CKAP4-STAT6-CHI3L3 axis, promoting CD8+ T-cell suppression, while its blockade restores neutrophil maturation, reduces tumor burden, and enhances PD-1 checkpoint responses, including complete responses with combination therapy [94,119]. Single-cell profiling of neuroblastoma bone marrow metastasis further identifies TAN subsets enriched for proangiogenic and immunosuppressive programs marked by VEGFA, PROK2, MMP9, and IL1RN, with a mature N-2 subset expressing CXCR1/CXCR2, MMP9, and CD177, suggesting roles in recruitment, matrix remodeling, angiogenesis, and immune escape [120]. These findings support a ‘reprogram rather than deplete’ strategy.

NETs provide a plausible mechanism by which neutrophils promote the outgrowth of dormant DTCs. NET-associated neutrophil elastase and MMP9 remodel laminin to generate an epitope that activates integrin α3β1 and downstream FAK/ERK/MLCK/YAP signaling, thereby driving dormant cancer cell reactivation in lung metastasis models [121]. While direct validation in bone dormancy models is still needed, MMP9, neutrophil elastase, and NET formation are recurring neutrophil effector programs that make this a plausible skeletal framework. The PAD4-CXCR2 axis may further link neutrophil trafficking and NETosis, as PAD4 regulates CXCR2 expression and chemotaxis, and its inhibition reduces neutrophil accumulation and metastasis while enhancing checkpoint blockade [122]. In bone, CXCL5-CXCR2 signaling is sufficient to shift cancer cells from quiescence to active colonization, and CXCR2 inhibition reduces this effect [106].

### 5.5. Bone Destruction/Formation Coupling: Neutrophils in the “Vicious Cycle”

#### 5.5.1. Osteoclast-Supportive Mechanisms

Within the skeletal metastatic niche, neutrophils may contribute to osteoclastogenesis through indirect inflammatory mechanisms rather than direct RANKL production. Activated neutrophils can release oncostatin M, IL-1, IL-6, and TNF-α, which induce RANKL expression in stromal and osteoblast-lineage cells, thereby promoting osteoclast formation and shifting the RANKL/OPG balance toward bone resorption [123]. These cytokine programs overlap with those that drive tumor invasion, immunosuppression, and endothelial activation, meaning that neutrophil-driven inflammation at the colonization front may simultaneously amplify osteolytic remodeling [95,96]. NET-associated proteases, including neutrophil elastase and MMP9, may further contribute by remodeling the extracellular matrix and altering stromal barriers in ways that plausibly support osteoclast-associated niche activation. However, direct validation of this mechanism in skeletal metastasis remains limited [124].

#### 5.5.2. Osteoblast-Lineage Effects

Neutrophils may also influence osteoblast-lineage cells, though this evidence is less developed and should not be overclaimed. Under specific conditions, neutrophil depletion increases bone mass and reduces marrow cavity size through direct neutrophil–osteoblast contact, suggesting that neutrophils can fine-tune osteoblast differentiation and endosteal niche architecture [125]. In contrast, mice lacking G-CSF or G-CSFR maintain normal bone structure despite profound neutropenia, indicating that neutrophils are not required for baseline skeletal homeostasis [123]. In the metastatic context, these findings raise the possibility that neutrophils may influence osteoblast-lineage programs, particularly in mixed or osteoblastic lesions such as those seen in prostate cancer. However, this remains speculative and requires direct experimental validation.

Neutrophils are not the core driver of the classical tumor-osteoblast-osteoclast-TGF-β vicious cycle; tumor-derived PTHrP, TGF-β, and direct tumor-stromal interactions primarily sustain that axis. However, neutrophils may amplify or modify this cycle depending on their maturation state, inflammatory context, and tumor type. In osteolytic disease, neutrophil-derived inflammatory mediators and proteolytic programs can reinforce osteoclast activation and matrix remodeling. In contrast, in osteoblastic or mixed lesions, their effects on osteoblast differentiation and local immune suppression may shift the balance differently. Thus, neutrophils should be viewed as context-dependent modifiers of the vicious cycle rather than its primary architects, with their contribution shaped by the evolving bone metastatic niche.

### 5.6. NETs as Adhesive, Proteolytic, and Immunosuppressive Scaffolds in Skeletal Colonization

NETs act as extracellular adhesive scaffolds that can physically capture CTCs, shielding them from peripheral immune surveillance. NET-associated DNA can be sensed by tumor-cell CCDC25, activating the ILK-β-parvin pathway to promote cancer-cell motility, and high CCDC25 expression is associated with poor prognosis [126]. NETs also suppress NK-cell and effector T-cell responses and cooperate with IL-17-producing γδ T cells to create a broadly immunosuppressive circulatory environment [109]. NET formation can be promoted by NADPH oxidase-derived ROS, and NETs may increase tumor-cell integrin α5β1 expression, thereby enhancing adhesion, proliferation, and migration [127]. Although most of this evidence comes from non-skeletal models, these mechanisms may still be relevant prior to skeletal colonization, as tumor cells must first survive in circulation, evade immune clearance, and establish stable endothelial interactions before reaching any distant niche, including the bone marrow.

#### 5.6.1. Vascular Arrest and Endothelial Permeability

NETs contribute to vascular arrest and extravasation by acting as thrombosis-like scaffolds that promote microvascular obstruction and disrupt the endothelial barrier. NET-associated β1-integrin engagement, together with NE- and MMP9-mediated degradation of endothelial tight junctions, converts CTC capture into vascular leakiness and transendothelial migration at metastatic sites [124]. NETs also enhance tissue factor expression and procoagulant activity in tumor cells, creating a thrombo-inflammatory environment that anchors CTCs to the vascular wall and supports metastatic seeding [128,129]. In postoperative and inflammatory metastasis models, NETs have been shown to promote tumor-cell extravasation, implantation, and proliferation [130]. Although such data are not bone-specific, they support the broader concept that NET-like vascular trapping and inflammatory occlusion mechanisms may facilitate metastatic arrest within marrow sinusoids, where adhesion molecules such as P-selectin, E-selectin, ICAM, and VCAM are constitutively expressed and may create a particularly permissive environment for NET-mediated anchoring.

#### 5.6.2. Dormancy Awakening

One of the most mechanistically defined NET functions in metastasis is their capacity to reactivate dormant DTCs. NET-associated NE and MMP9 remodel laminin in the extracellular matrix, generating a proteolytic epitope that activates integrin α3β1 and downstream FAK/ERK/MLCK/YAP signaling, driving dormant cancer cells back into active proliferation [121]. Chemotherapy-induced fibroblast senescence can further promote NET formation, providing an inflammation-driven trigger for dormancy escape [131]. While this mechanism has been demonstrated primarily in lung metastasis models, MMP9, NE, NET formation, and matrix remodeling are recurring neutrophil effector programs that make it a plausible framework for awakening skeletal dormancy, pending direct validation in bone-specific models.

## 6. Neutrophil-Lineage Suppressor Cells and Immunotherapy Resistance in Skeletal Metastasis

### 6.1. PMN-MDSCs Within the Neutrophil-Lineage Spectrum: Operational Definitions and Pitfalls

PMN-MDSCs should not be viewed as a completely separate cell type from neutrophils, but rather as a functionally defined, immunosuppressive state within the broader neutrophil-lineage spectrum [132,133]. They overlap extensively with conventional neutrophils in morphology and surface marker expression but are distinguished operationally by their ability to suppress T-cell responses through mechanisms such as arginase-1 activity, reactive oxygen species generation, checkpoint ligand expression, and immunosuppressive cytokine production. PMN-MDSCs may support skeletal colonization by promoting pre-metastatic niche formation, suppressing anti-tumor immunity, remodeling the extracellular matrix, and enhancing metastatic outgrowth [14,134]. Despite this overlap, PMN-MDSCs are more consistently associated with immunosuppression and tumor progression, whereas neutrophils display greater functional plasticity and can sometimes support anti-tumor immunity [16,132]. When comparing primary tumors and metastatic stroma, PMN-MDSCs are more infiltrated in the metastatic stroma [135].

There is a lack of unique surface markers to distinguish MDSCs from classical neutrophils, and functional validation in human cancer is challenging. In addition, translational barriers arise from differences between murine models and human patients [136].

### 6.2. Bone Metastasis as a Site Enriched for Immature/Suppressive Myeloid States

Bone metastatic environments are notably enriched with MDSCs, particularly PMN-MDSCs, which are recruited by tumor-derived factors and local signals within the bone microenvironment [137,138]. These cells exert potent immunosuppressive effects, dampening anti-tumor immune responses and facilitating tumor progression [132]. For example, studies in breast cancer and multiple myeloma have shown that PMN-MDSCs accumulate in the bone marrow, where they not only suppress T cell activity but also promote bone destruction and chemoresistance, thereby contributing to the failure of immunotherapies and conventional treatments [133,139]. In addition, MDSCs can differentiate into osteoclasts, directly contributing to bone destruction and osteolytic lesions. Tumor-derived exosomes promote MDSC recruitment and differentiation, further enhancing immunosuppression and bone metastasis [137].

**Figure 3:**
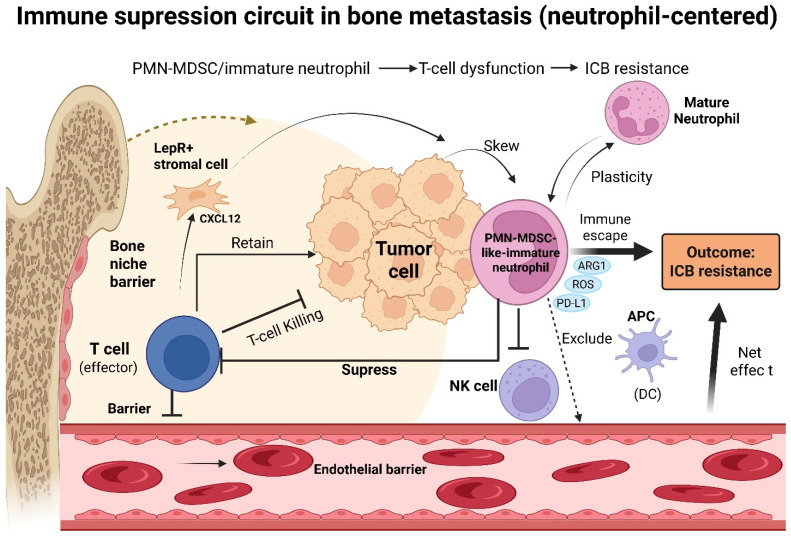
Neutrophil-centered immune suppression circuit in bone metastasis. Conceptual model showing how immature neutrophils and PMN-MDSC-like neutrophil-lineage cells may contribute to immune escape and immune checkpoint blockade (ICB) resistance in bone metastasis. Within the metastatic marrow niche, stromal CXCL12, tumor-derived factors, and inflammatory signals can support retention, recruitment, and functional skewing of neutrophil-lineage cells. Mature neutrophils may retain cytotoxic potential in some contexts, but tumor progression can drive plasticity toward immature or PMN-MDSC-like states. These suppressive cells inhibit effector T-cell and NK-cell activity through mechanisms that include ARG1, ROS, PD-L1-associated checkpoint signaling, and physical or functional immune exclusion. The resulting T-cell dysfunction, impaired cytotoxicity, and altered antigen-presenting cell activity create a permissive immune environment that favors tumor persistence and contributes to ICB resistance. This model emphasizes that therapeutic strategies should focus on selectively inhibiting or reprogramming pathogenic neutrophil states rather than indiscriminately depleting neutrophils. Abbreviations: PMN-MDSC, polymorphonuclear myeloid-derived suppressor cell; ICB, immune checkpoint blockade; NK cell, natural killer cell; ARG1, arginase 1; ROS, reactive oxygen species; PD-L1, programmed death ligand 1; APC, antigen-presenting cell. Created in BioRender. Mohammad, K. (2026) https://BioRender.com/yvcogza (accessed on 29 June 2026).

Complete depletion of myeloid-derived suppressor cells (MDSCs) remains challenging and may produce undesirable effects because related myeloid populations also participate in physiological host defense and tissue repair. Therefore, selectively targeting pathogenic molecules, trafficking pathways, or suppressive functions may be more feasible than broad depletion [140]. Standard bone-targeted therapies may intersect with neutrophil-lineage biology mainly by altering the bone-remodeling niche rather than by directly targeting neutrophils. Bisphosphonates, denosumab, and radiopharmaceuticals such as Radium-223 are clinically important bone-directed approaches, but their direct effects on NET formation, neutrophil state, or PMN-MDSC recruitment in skeletal metastasis remain insufficiently defined [10]. This intersection is biologically plausible because osteoclast-mediated bone resorption releases matrix-stored growth factors, including TGF-β, and sustains the tumor–osteoblast–osteoclast vicious cycle, while immune and myeloid populations shape osteoclastogenesis, stromal inflammation, and bone destruction [40,41,137,139]. Antiresorptive treatments alone are insufficient in the context of elevated granulocytic populations, suggesting that effective management of established bone metastases may require a combinatorial strategy targeting both granulocytes and osteoclasts. In a recent study, using a murine model of bone metastasis resistant to osteoclast inhibition by antiresorptive agents such as zoledronic acid (ZA), they evaluated the impact of MDSC depletion via anti-Gr1 treatment in mice bearing skeletal tumors. antiresorptive agents such as zoledronic acid (ZA) [141]. These findings suggest that neutrophil- or PMN-MDSC-directed strategies may be most effective when added to standard bone-targeted therapy rather than used as replacement. A neutrophil-centered model of immune suppression and ICB resistance in bone metastasis is illustrated in Figure 3.

### 6.3. Therapeutic Implications

Emerging therapeutic strategies in cancer treatment include pharmacological inhibition of tumor-derived factors that mediate neutrophil recruitment and polarization, as well as selective targeting of neutrophil pro-tumorigenic functions. These approaches can be integrated with conventional or novel anticancer therapies to enhance overall therapeutic efficacy [142].

Targeting specific molecules that inhibit the immunosuppressive properties of PMN-MDSCs is another approach that could inhibit tumor growth. Examples include using chemokine inhibitors such as CCR2 blockers, CXCR2 blockade, arginase inhibitors, or STAT3 pathway blockers [137]. Combination therapy with CXCR2 antagonists (e.g., SX-682) and immune checkpoint inhibitors (anti-PD1) significantly reduced tumor growth and improved survival compared to single-agent treatments [143].

Therapeutically, targeting PMN-MDSCs in bone metastasis offers promising avenues to overcome immunotherapy resistance. Strategies include inhibiting their recruitment, blocking their immunosuppressive functions, or reprogramming them toward a less suppressive phenotype [137,138]. By specifically targeting PMN-MDSCs, it may be possible to reduce tumor-induced bone destruction, improve patient responses to immunotherapy, and ultimately achieve better clinical outcomes in bone metastatic cancers [132,139].

## 7. Tumor-Type Lenses

### 7.1. Breast Cancer Bone Metastasis

Breast cancer bone metastasis is commonly associated with osteolytic lesions driven by reciprocal interactions between tumor cells, osteoclasts, osteoblast-lineage cells, and the bone marrow microenvironment. Breast cancer cells promote osteoclastogenesis via factors including IL-6, IL-8/CXCL8, IL-11, PTHrP, TGF-β, GM-CSF, and G-CSF, thereby enhancing stromal RANKL signaling and osteoclast differentiation [144,145,146,147,148]. Osteoclast-mediated bone resorption then releases matrix-bound growth factors, including TGF-β, further supporting tumor growth and osteolytic progression [149,150,151].

Within this skeletal niche, neutrophil involvement appears most relevant at the intersection of tumor-intrinsic programs and marrow trafficking and inflammatory chemokine pathways. In triple-negative breast cancer, CTNND1 silencing increased bone metastatic capacity and was associated with activation of the CXCR4/CXCL12 axis, enhanced bone homing, increased neutrophil infiltration in bone, and upregulation of neutrophil-relevant cytokines, including GM-CSF and IL-8/CXCL8 [100]. This provides a bone metastasis-specific link between tumor-cell signaling, marrow tropism, and neutrophil-rich skeletal colonization.

The CXCL5/CXCR2 axis has also been implicated in breast cancer bone colonization, connecting tumor-derived chemokine signaling with myeloid recruitment and metastatic niche formation [152]. Similarly, S100A14-driven induction of CCL2 and CXCL5 through RAGE/NF-κB signaling suggests that breast cancer cells can generate inflammatory chemokine programs that support myeloid cell recruitment during metastasis [113]. However, CXCR2 biology remains context-dependent, and whether CXCR2-driven neutrophil recruitment or reprogramming has the same functional consequences in bone as in other metastatic sites remains unresolved. This is particularly important because neutrophil localization in bone reflects a balance between CXCR2-mediated mobilization and CXCR4/CXCL12-dependent marrow retention [153,154].

### 7.2. Prostate Cancer Bone Metastasis

Prostate cancer bone metastases represent a complex pathophysiological process involving multiple cellular interactions beyond simple osteoblastic activity. These metastases are characterized by abnormal osteoblast activation, persistent osteoclast–osteoblast coupling, increased bone turnover, endothelial remodeling, immune suppression, and marrow niche reprogramming [155,156]. In this setting, neutrophils may influence skeletal metastasis through mechanisms distinct from those seen in predominantly osteolytic tumors. Rather than primarily amplifying osteoclast-driven bone destruction, they may regulate tumor-cell survival, marrow retention, motility, immune escape, and adaptation to the bone niche [20,90].

Available evidence suggests that neutrophil function in prostate cancer bone metastasis is stage- and therapy-dependent. In early disease, bone marrow neutrophils can induce apoptosis of prostate cancer cells by suppressing STAT5 signaling, indicating a potential anti-tumor role [95]. During progression, however, tumor cells can become resistant to neutrophil-mediated killing, and androgen deprivation therapy may further weaken neutrophil anti-tumor activity through increased TβRI signaling; this effect can be reversed by TβRI inhibition [157].

Neutrophils may also support skeletal tropism through protease-mediated signaling. Neutrophil-derived proteinase 3, either secreted or displayed on the neutrophil surface, can bind RAGE on prostate cancer cells and activate ERK1/2 and JNK1 signaling, increasing tumor-cell motility and promoting homing toward the proteinase 3-rich bone marrow environment [112]. Thus, prostate cancer illustrates a context-dependent model in which neutrophils may contribute to both anti-tumor immunity and metastatic support, with the dominant effect shaped by disease stage, androgen signaling, therapy exposure, and the evolving marrow niche.

### 7.3. Lung Cancer and Other Bone-Tropic Primary Tumors

Lung cancer, particularly NSCLC, is a frequent source of skeletal metastasis. NSCLC bone lesions are often osteolytic and are driven by interactions between tumor cells, osteoclast-lineage cells, and the marrow microenvironment. Several tumor-derived pathways can promote osteoclastogenesis, including CXCR4-dependent induction of soluble VCAM1, exosomal amphiregulin-mediated activation of EGFR signaling in pre-osteoclasts, and LIGHT/TNFSF14-driven stimulation of osteoclast precursors [158,159,160]. Although neutrophil-associated programs such as TAN accumulation, NET formation, protease release, PD-L1-associated immunosuppression, and immunotherapy resistance have been described in lung cancer, their specific roles in lung cancer bone metastases remain poorly defined [161,162,163].

Compared with breast and prostate cancer, direct evidence for neutrophil-specific regulation of lung, renal, thyroid, gastrointestinal, melanoma, and endocrine tumor bone metastases remains sparse. These tumors may share general neutrophil programs, CXCR1/2 recruitment, NET formation, protease release, ROS, and PMN-MDSC-like suppression, but the field lacks tumor-type-specific skeletal studies. Therefore, these tumor types should be presented primarily as evidence gaps rather than as established models of neutrophil-driven skeletal metastasis.

## 8. Clinical and Translational Evidence

### 8.1. Circulating Neutrophil-Associated Biomarkers

Circulating biomarkers offer a minimally invasive, although indirect, view of neutrophil-related activity in metastatic cancer. The neutrophil-to-lymphocyte ratio (NLR) is the most widely validated marker, reflecting the balance between systemic inflammation and adaptive immune function. Elevated NLR is consistently linked to advanced disease, greater metastatic burden including bone involvement, and poorer survival [164]. In patients with bone metastases, NLR independently predicts overall survival, supporting its clinical utility for prognostication in this setting. Mechanistically, this reflects the expansion of neutrophil populations alongside relative lymphocyte depletion, indicative of a shift toward myeloid-driven immunosuppression [165].

Importantly, the clinical value of NLR extends beyond baseline measurement. Longitudinal changes in NLR have been associated with recurrence and metastatic progression, highlighting its potential as a dynamic biomarker for disease monitoring [166]. However, its interpretation remains limited by variability in cut-off thresholds across studies and susceptibility to confounding factors such as infection, treatment-induced inflammation, and corticosteroid exposure [167]. As such, NLR should be viewed as a robust but biologically non-specific indicator of systemic immune dysregulation rather than a direct measure of neutrophil function [164].

More mechanistically informative circulating biomarkers are linked to neutrophil extracellular trap (NET) formation. NET-associated components, including cell-free DNA (cfDNA), myeloperoxidase (MPO) DNA complexes, neutrophil elastase, and citrullinated histone H3 (Cit-H3), have been detected in patient plasma and are increasingly explored as surrogates of neutrophil activation states [168]. These markers have been associated with metastatic progression, pre-metastatic niche formation, and immune evasion, including T cell dysfunction mediated by NET-associated immunosuppressive signaling [168,169]. Clinically, circulating NET markers, such as MPO–DNA complexes, are elevated in metastatic settings, whereas Cit-H3 has been proposed as a more specific biomarker associated with minimal residual disease and bone marrow involvement. Nevertheless, translation into routine clinical use remains limited by pre-analytical variability, lack of assay standardization, and overlap with non-neutrophil sources of circulating DNA [169].

### 8.2. Tissue and Spatial Biomarkers of Neutrophil Infiltration and Functional State

At the tissue level, direct characterization of neutrophils within bone metastases remains challenging due to the limited accessibility of skeletal lesions. Bone metastases are not routinely biopsied, and available samples are often small, decalcified, and spatially restricted, limiting comprehensive molecular profiling [170]. Nevertheless, tissue-based biomarkers provide more direct insight into neutrophil localization, activation state, and functional interactions within the tumor microenvironment [142]. Immunohistochemical and spatial analyses commonly evaluate neutrophil infiltration using markers such as CD66b, myeloperoxidase (MPO), and neutrophil elastase (NE), which identify neutrophil abundance and activation within metastatic lesions [171,172,173]. Increased infiltration of CD66b^+^ neutrophils has been associated with immunosuppressive microenvironments, metastatic progression, and adverse clinical outcomes across multiple cancers [173,174].

Functional characterization of tumor-associated neutrophils increasingly incorporates markers associated with suppressive and pro-tumorigenic phenotypes [142]. Arginase-1 (ARG1) and PD-L1 expression have been linked to neutrophil-mediated T-cell suppression and immune evasion, particularly within highly inflammatory metastatic niches [175,176]. Because ARG1 and PD-L1 are shared across several myeloid populations, they should be interpreted together with neutrophil markers such as CD66b, MPO, NE, or spatial co-localization rather than used alone.

Similarly, chemokine receptors such as CXCR2 and CXCR4 provide insight into neutrophil trafficking dynamics and interactions with the bone marrow niche [177]. CXCR2 is primarily associated with inflammatory neutrophil recruitment, whereas CXCR4 is linked to bone marrow retention, metastatic homing, and stromal niche signaling within skeletal metastases [178].

Recent advances in single-cell RNA sequencing (scRNA-seq) and spatial transcriptomics have enabled higher-resolution mapping of immune and stromal heterogeneity within metastatic lesions [66]. These approaches have revealed complex cellular ecosystems involving diverse myeloid populations, stromal interactions, and dynamic cell–cell communication networks that support metastatic progression [23]. Importantly, single-cell analyses demonstrate substantial differences between primary tumors and metastatic lesions, including enhanced immunosuppressive signaling and altered spatial organization in metastatic bone disease [179,180]. Spatial profiling additionally enables visualization of neutrophil localization relative to tumor cells, vasculature, and stromal compartments, thereby improving understanding of niche-specific interactions that facilitate tumor colonization and immune escape [181].

Spatial assessment of NET formation within tissues is also increasingly recognized as clinically relevant [182]. Detection of citrullinated histone H3 (Cit-H3), often combined with MPO or NE co-localization, is widely used to identify NET structures in situ and has been associated with metastatic dissemination, thrombosis, and therapy resistance [168,169]. However, these technologies retain important limitations in the context of neutrophil biology. Neutrophils are short-lived, transcriptionally low, and highly sensitive to tissue processing, resulting in their frequent underrepresentation in single-cell datasets [183]. Enzymatic dissociation protocols may preferentially deplete granulocytes, while spatial platforms may lack sufficient marker specificity to distinguish closely related myeloid subsets [184,185]. Consequently, current tissue-based approaches likely underestimate the abundance and functional diversity of neutrophils within metastatic bone lesions. Integration of spatial profiling with proteomics, imaging approaches, and circulating biomarkers will therefore be essential to achieve a more comprehensive understanding of neutrophil-driven mechanisms within the metastatic bone niche [186].

## 9. Therapeutic Landscape and Actionable Axis

### 9.1. Block Recruitment

Blocking neutrophil recruitment is one of the most actionable therapeutic strategies because tumor-derived chemokines can sustain a continuous influx of neutrophils and polymorphonuclear myeloid-derived suppressor cells (PMN-MDSC) into the tumor microenvironment [143]. CXCR2 is particularly relevant because it is highly expressed by neutrophils and mediates chemotaxis in response to CXC ligands such as CXCL1, CXCL2, CXCL5, and CXCL8/IL-8 [178]. In cancer, CXCR2 contributes to tumor progression through recruitment of MDSCs and tumor-associated neutrophils (TANs), pre-metastatic niche formation, angiogenesis, and, in some contexts, direct tumor-cell signaling [178].

CXCR2 antagonism has therefore been proposed as a strategy to reduce suppressive myeloid trafficking and improve T-cell-mediated anti-tumor immunity [72]. Preclinical studies show that CXCR1/CXCR2 blockade with agents such as SX-682 can reduce intratumoral MDSCs, increase CD8^+^ T-cell infiltration, and enhance response to anti-PD-1 therapy in breast cancer models [178]. However, CXCR2 signaling is context-dependent, and CXCR2 loss can paradoxically accelerate metastasis by shifting TANs toward a pro-tumorigenic TAN2 phenotype with impaired cytotoxicity and enhanced angiogenesis [178,187]. Similarly, inhibition of CXCR2-dependent MDSC trafficking has been shown to enhance T-cell immunotherapy, supporting its use as a rational partner for immune checkpoint blockade and adoptive cell therapy [143].

However, CXCR2 blockade should not be framed only as simple “neutrophil exclusion.” CXCR2 is not restricted to neutrophils, and its expression in other stromal, endothelial, immune, and tumor-associated compartments complicates the interpretation of inhibitor studies [178,188]. Moreover, recent evidence indicates that CXCR2 antagonism may slow tumor growth without fully preventing TAN recruitment; instead, it can impair neutrophil polarization toward immunosuppressive phenotypes and reduce lymphocyte suppression by decreasing reactive oxygen species and arginase-1 release [188]. This distinction is important for clinical translation because it suggests that therapeutic benefit may arise as much from functional reprogramming of neutrophils as from reduced trafficking [72].

CXCR4/CXCL12 modulation represents a related but biologically distinct axis, particularly relevant to skeletal metastasis, as CXCL12-rich bone marrow niches regulate cell retention, homing, and mobilization [189,190]. In breast cancer, the CXCR4/CXCL12 axis provides directional cues for organ-specific metastasis, and CXCR4 has been reported as upregulated in bone-metastasized breast cancer cells [191]. In prostate cancer bone metastasis models, CXCR4 signaling contributes to docetaxel resistance, while CXCR4 inhibition with balixafortide enhanced docetaxel-mediated anti-tumor activity [192].

Importantly, CXCR4/CXCL12 should be positioned as a bone-niche intervention rather than a straightforward neutrophil-recruitment blocker [193]. Balixafortide and related CXCR4 inhibitors can mobilize hematopoietic stem cells from the marrow, and this concept has been proposed to dislodge cancer stem-like cells from protective bone marrow niches and increase chemotherapy accessibility [192,194]. Therefore, targeting CXCR4/CXCL12 may simultaneously affect tumor cells, immune cells, hematopoietic cells, and stromal niche interactions, making therapeutic timing and biomarker-guided patient selection essential [177].

Overall, targeting recruitment pathways should be conceptualized as modulating chemokine-driven trafficking and niche retention rather than as a global depletion of neutrophils, with CXCR2 primarily regulating inflammatory recruitment and CXCR4/CXCL12 governing bone marrow niche biology [178,195].

### 9.2. Block Effector Programs: NETosis, Protease Release, and Redox-Mediated Suppression

Targeting neutrophil effector programs represents a strategy to disrupt the downstream mechanisms by which neutrophils promote tumor progression while preserving their essential role in host defense [179]. NETs are central mediators of these effects, functioning as extracellular DNA scaffolds enriched with histones, neutrophil elastase, and other granule-derived proteins that support tumor progression and metastatic dissemination [97,179]. In cancer, NETs enhance circulating tumor cell (CTC) survival, adhesion, and extravasation, thereby promoting metastatic spread [104].

NET formation is mechanistically heterogeneous, involving both PAD4-dependent chromatin decondensation and alternative PAD4-independent pathways, which may limit the efficacy of single-target inhibition strategies [97,168]. Therapeutic approaches to NET inhibition include degradation of extracellular DNA using DNase, as well as upstream targeting of signaling pathways, such as ROS–PAD4-mediated NETosis [97]. Experimental evidence demonstrates that inhibition of the ROS–PAD4 axis suppresses NET formation and reduces metastatic burden without affecting neutrophil abundance [196].

Beyond DNA scaffolds, NET-associated components such as histones and neutrophil proteases also contribute to tumor-promoting inflammation, extracellular matrix remodeling, and immune modulation [77,97]. NETs also play a role in cancer-associated thrombosis and immunothrombosis, linking coagulation pathways to metastatic niche stabilization and disease progression [97]. The functional impact of NETs is also temporally dynamic, contributing to both early pre-metastatic niche formation and later stages of immune evasion and therapeutic resistance [104].

Protease inhibition is a complementary strategy for targeting neutrophil effector functions. Neutrophil-derived proteases, including neutrophil elastase, cathepsin G, proteinase 3, and matrix metalloproteinase, promote tumor progression through extracellular matrix remodeling, activation of proliferative signaling pathways, and facilitation of tumor invasion [77,79]. Neutrophil elastase is particularly important, as it is released during both degranulation and NETosis and has been implicated in tumor growth and metastasis across multiple cancer types [172,174]. Evidence from primary bone tumors, such as osteosarcoma, may provide mechanistic clues about neutrophil proteases and NETs, but it should not be taken as direct evidence of skeletal metastasis [197].

NET-associated protease activity also contributes to tumor vascularization and metastatic progression, as demonstrated in osteosarcoma models, in which neutrophil recruitment and NET formation enhance angiogenesis and lung metastasis [198]. However, targeting proteases requires caution due to significant functional redundancy among neutrophil-derived enzymes, which may limit the efficacy of single-agent inhibition strategies [172]. Moreover, these proteases are essential for antimicrobial defense and physiological inflammatory responses, raising concerns regarding potential off-target effects and infection risk [79,172].

ROS/RNS production is a distinct neutrophil effector program that can act independently of NETosis. Beyond supporting NET formation, neutrophil-derived ROS/RNS can suppress T-cell signaling and proliferation, promote immune dysfunction, and modify stromal inflammation. In skeletal metastasis, these effects may contribute to osteoclast-linked inflammation, endothelial activation, and immune exclusion, although direct bone-specific evidence remains limited. Thus, redox-targeted approaches may limit both NET-dependent and NET-independent tumor support but must be used cautiously because oxidative burst is essential for antimicrobial defense.

Despite strong mechanistic and preclinical evidence, clinical validation of NET- and protease-targeted therapies remains limited, particularly in the context of bone metastasis [97,199]. Overall, blocking of neutrophil effector programs should be considered a precision strategy to selectively inhibit pro-tumorigenic functions, such as NETosis and protease activity, ideally in combination with immunotherapy or standard-of-care treatments and guided by biomarker-driven patient selection [104].

Together, these considerations support a broader effector-program framework in which NETs, proteases, and ROS/RNS function as overlapping yet therapeutically distinct neutrophil outputs.

### 9.3. Reprogram Neutrophil State

Reprogramming neutrophil functional states has emerged as a conceptual therapeutic strategy to shift neutrophils from suppressive or immature phenotypes toward inflammatory and cytotoxic anti-tumor states, rather than eliminating them entirely [23]. Neutrophils exhibit profound phenotypic heterogeneity and plasticity in cancer, with tumor-associated neutrophils (TANs) capable of adopting both pro-tumorigenic and anti-tumorigenic states depending on microenvironmental cues [200]. This functional spectrum is often described along an N1–N2 continuum, in which N1-like neutrophils exhibit cytotoxic and immunostimulatory properties, whereas N2-like neutrophils promote immunosuppression, tumor growth, and metastasis [201].

Despite this promise, reprogramming strategies remain largely conceptual and face significant translational challenges. A major limitation is the lack of definitive markers to distinguish functionally distinct neutrophil subsets in patients, complicating both therapeutic targeting and biomarker development [200]. Furthermore, neutrophil plasticity is highly context-dependent and influenced by tumor type, disease stage, and treatment exposure, necessitating careful consideration of timing and therapeutic combinations [201].

### 9.4. NET-Targeted Therapy Concepts

Several therapeutic strategies targeting NETs have shown preclinical promise, though direct clinical evidence in bone metastasis remains limited and claims should be interpreted cautiously. DNase I-mediated NET degradation has been shown to enhance anti-PD-1 efficacy by increasing CD8+ T-cell infiltration and cytotoxicity in tumor models, suggesting that NETs contribute to immune checkpoint resistance and that their degradation may restore immunotherapy responsiveness [202]. PAD4 inhibition represents a more upstream strategy; genetic deletion or pharmacologic blockade of PAD4 reduces histone citrullination, NET formation, neutrophil accumulation, and metastasis while enhancing checkpoint blockade responses [122]. Because PAD4 also regulates CXCR2 expression and neutrophil chemotaxis, PAD4 inhibition may simultaneously reduce neutrophil trafficking and NETosis, positioning it as a dual-target approach. ROS inhibition, particularly via NADPH oxidase blockade, can suppress suicidal NETosis and has shown additive effects when combined with integrin α5β1 inhibition to reduce NET-driven tumor progression in vivo [127]. CXCR2 blockade represents another upstream strategy, reducing neutrophil and PMN-MDSC recruitment to tumors and premetastatic niches and thereby limiting the pool of NET-competent neutrophils available at metastatic sites [106,178]. Combining NET-targeted strategies with anti-PD-1 or anti-PD-L1 checkpoint blockades appears particularly rational given that NETs suppress cytotoxic T-cell activity and that NET degradation or PAD4 inhibition consistently improves checkpoint response in preclinical models. Whether these combinations translate to improved outcomes in skeletal metastasis specifically remains an open question that warrants dedicated investigation.

### 9.5. Translational Caution

Despite promising preclinical evidence, important translational limitations continue to complicate the clinical development of neutrophil-targeted therapies [67]. Neutrophils remain essential mediators of antimicrobial defense and tissue repair; therefore, excessive suppression of neutrophil recruitment or effector activity may increase susceptibility to infection and impair wound healing, particularly in patients receiving chemotherapy, radiotherapy, or surgical intervention [203]. In addition, neutrophil biology is characterized by substantial functional plasticity, with neutrophils capable of adopting both tumor-promoting and anti-tumor phenotypes depending on disease stage, inflammatory context, and therapeutic exposure. Consequently, indiscriminate neutrophil depletion may unintentionally compromise beneficial inflammatory or cytotoxic immune responses [204]. Recent evidence further indicates that neutrophils may exert distinct functions during early metastatic colonization compared with established metastatic lesions, in which their roles shift toward maintaining immunosuppression, angiogenesis, stromal remodeling, and therapeutic resistance [142,203]. These temporal differences suggest that therapeutic timing and disease-stage stratification are likely to be critical determinants of treatment efficacy [67]. Furthermore, the lack of standardized and clinically validated biomarkers remains a major barrier to patient selection and treatment monitoring [13]. Biomarker-guided strategies incorporating circulating NET-associated markers, neutrophil-related transcriptional signatures, and tissue-based spatial profiling may therefore be necessary to identify patients most likely to benefit from neutrophil-directed interventions while minimizing off-target toxicity and unnecessary immune suppression [13,204]. Overall, successful translation will likely require context-specific modulation of neutrophil function rather than broad suppression approaches. Representative clinical trials and clinical-stage strategies targeting neutrophil-lineage trafficking, PMN-MDSC-associated pathways, or NET-related biology are summarized in Table 1.

This table is representative rather than exhaustive. Most clinical trials target neutrophil-lineage biology indirectly through CXCR1/2 signaling, MDSC modulation, or myeloid reprogramming rather than through bone-specific neutrophil depletion. Direct NET-targeted clinical trials in skeletal metastasis remain lacking, highlighting an important translational gap.

Abbreviations: ATRA, all-trans retinoic acid; CRPC, castration-resistant prostate cancer; CXCR, C-X-C chemokine receptor; HDAC, histone deacetylase; MDSC, myeloid-derived suppressor cell; MSS, microsatellite stable; NET, neutrophil extracellular trap; NSCLC, non-small cell lung cancer; OS, overall survival; PAD4, peptidyl arginine deiminase 4; PD-1, programmed cell death protein 1; PMN-MDSC, polymorphonuclear myeloid-derived suppressor cell; rPFS, radiographic progression-free survival; TME, tumor microenvironment.

## 10. Methods/Technology Toolbox

### 10.1. Experimental Models

Animal models are essential for studying bone metastasis, and commonly used approaches include tail vein, intracardiac, intraosseous, orthotopic, and tail artery injection models. Intracardiac injection is widely used because it rapidly produces detectable metastases suitable for bioluminescence imaging, while intraosseous injection generates bone lesions quickly but disrupts bone integrity; orthotopic models best mimic the full metastatic cascade, and tail artery injection provides a simple, efficient route to promote hind-limb bone metastases [205].

Bone metastasis models use different mouse strains depending on the research goal. Immunodeficient mice (e.g., nude, SCID, NOD/SCID) are mainly used for xenograft and humanized models, enabling engraftment of human tumor cells but limiting the study of immune interactions. In contrast, immunocompetent strains (e.g., C57BL/6, BALB/c) allow investigation of immune responses and spontaneous metastasis, though they typically rely on murine tumor cells [206]. In addition, other animal model are used to study metastasis, including autochthonous models, genetically engineered mouse models (GEMMs), transplant models, and human-in-mouse systems such as PDXs and CDXs. Autochthonous models and GEMMs allow investigation of tumor development within an intact immune system, whereas transplant models enable rapid and reproducible tumor growth. Humanized models better preserve tumor heterogeneity and are useful for biomarker discovery and drug testing [207].

Besides the challenges in modeling bone metastasis, there are challenges in modeling human neutrophils in animal models. Traditional humanized mouse models do not support mature, functional human neutrophils in circulation or tissues, limiting the study of human neutrophil biology in vivo [208]. In addition, human and mouse neutrophils differ in their cytokine profiles. For example, human neutrophils do not produce IFNβ, IL-10, or IL-17, while murine neutrophils do. This alters the inflammatory milieu and immune response [209]. These disparities help explain why mouse findings do not always translate directly to human biology and why humanized models are important for studying functional human neutrophils in vivo [208].

### 10.2. Single-Cell and Spatial Limitations for Neutrophils

Recent research has revealed that neutrophils are far more heterogeneous than previously thought, displaying many transcriptional and phenotypic states, although only a few of these have known functions. Understanding the global architecture of neutrophils may help in the development of neutrophils as therapeutic targets. In a study using single-cell RNA sequencing (scRNA-seq) to profile neutrophils across diverse tissues, conditions, and biological variables, generating an integrated transcriptional atlas termed NeuMap. This map reveals the global diversity of neutrophils and organizes them into seven functional transcriptional hubs linked to processes such as proliferation, immunosuppression, and angiogenesis, which were validated experimentally. Importantly, neutrophils form a continuous transcriptional landscape rather than distinct subpopulations, reflecting their dynamic and rapidly renewing nature. NeuMap provides a unifying framework for understanding neutrophil heterogeneity across diseases, highlighting that targeting transitions between functional states may offer more effective therapeutic strategies than focusing solely on terminal neutrophil subsets [210]. A multi-modal study of metastatic breast cancer analyzed 67 biopsies from 60 patients across multiple anatomical sites using single-cell and single-nucleus RNA sequencing, combined with spatial profiling techniques (e.g., Slide-seq, MERFISH, CODEX). By integrating these datasets with histopathology, the study mapped cell types, gene expression patterns, immune features, and spatial organization within the tumor microenvironment. This approach enabled a comprehensive characterization of metastatic niches and highlighted the complementary strengths of different profiling technologies [211].

Neutrophils are frequently underestimated in scRNA-seq datasets because they are fragile and transcript-poor, and spatial approaches may partly overcome this by preserving in situ context and minimizing dissociation-related loss. For solutions, the best evidence-backed points are rapid, fresh processing; neutrophil-conscious QC/analysis settings; enrichment-free workflows when possible; and integration of spatial omics with single-cell data [212,213].

### 10.3. Causal Inference: What Qualifies as “Neutrophil-Driven” in Bone Metastasis?

Demonstrating that a phenotype is truly “neutrophil-driven” in bone metastasis requires more than standard depletion studies. Anti-Gr1, an antibody that depletes neutrophils, has important limitations in specificity and can induce compensatory granulopoiesis [214]. These issues are especially relevant in bone, where anti-Gr1 has been reported to cause paradoxical accumulation of osteoclastogenic myeloid cells rather than reducing metastasis [141]. Therefore, stronger causal evidence should come from state-specific approaches, such as CXCR2 blockade, PAD4 inhibition, or adoptive transfer of defined neutrophil subsets, combined with functional validation [215,216].

## 11. Key Controversies and Open Questions

Despite growing interest in neutrophils as regulators of bone metastasis, several unresolved issues continue to limit interpretation of the field. The first question is whether neutrophils are true drivers of skeletal colonization or mainly secondary responders to tumor-induced inflammation. Many studies show that neutrophils or neutrophil-associated signatures increase during metastatic progression, but such associations alone do not prove causality [13,67]. This distinction is especially important in bone, where tumor growth, osteolysis, marrow remodeling, and emergency granulopoiesis occur together and can all increase neutrophil recruitment or alter neutrophil phenotype [11,141].

A second controversy concerns the dual role of neutrophils. In some settings, particularly early during metastatic colonization, neutrophils may retain cytotoxic or immune-surveillance functions. In other contexts, especially during established disease, they acquire immature, suppressive, or PMN-MDSC-like phenotypes that support tumor outgrowth and resistance to therapy. This stage-dependent behavior argues against describing neutrophils as uniformly anti-tumor or pro-tumor. Instead, their function should be interpreted according to tumor type, metastatic stage, treatment exposure, maturation state, and the immune composition of the marrow niche [13,86].

A third unresolved issue concerns the relationships among neutrophils, TANs, low-density neutrophils, immature neutrophils, and PMN-MDSCs. These populations overlap in morphology and surface-marker expression, particularly in the bone marrow, where developing granulocytic cells are abundant. PMN-MDSCs are best defined by suppressive function rather than by phenotype alone, yet many studies still rely mainly on marker-based identification [68,71]. This creates uncertainty when comparing mouse and human studies and when assigning specific functions to neutrophil-lineage subsets within metastatic bone lesions.

NET biology raises another major question. NETs can trap circulating tumor cells, remodel extracellular matrix, alter vascular permeability, suppress anti-tumor immunity, and awaken dormant tumor cells in non-skeletal models [119,121]. These mechanisms are biologically plausible in bone, where marrow sinusoids, inflammatory niches, and matrix remodeling provide a permissive environment for tumor-cell arrest and outgrowth. However, direct evidence that NETs awaken dormant disseminated tumor cells specifically within bone remains limited. Bone-specific models are needed to determine whether NET-mediated laminin remodeling, integrin activation, and downstream proliferative signaling operate in skeletal dormancy [106,121].

Another important gap is whether neutrophils behave differently in osteolytic, osteoblastic, and mixed lesions. In osteolytic metastases, neutrophil-derived cytokines, proteases, ROS, and NET-associated enzymes may amplify osteoclastogenesis and matrix destruction [95,96]. In osteoblastic or mixed lesions, particularly in prostate cancer, neutrophils may instead influence tumor-cell motility, marrow retention, osteoblast-lineage activity, immunosuppression, and therapy adaptation [95,157]. This distinction is conceptually important but remains insufficiently tested in direct comparative studies.

Therapeutically, it remains unclear whether the best strategy is to deplete neutrophils, block their recruitment, inhibit specific effector programs, or reprogram suppressive neutrophil-lineage states. Broad depletion may be ineffective or harmful because neutrophils are essential for host defense and may retain anti-tumor activity in some contexts. In bone metastasis, depletion strategies may also indirectly affect osteoclast precursors, hematopoietic progenitors, and other myeloid populations. More selective approaches, such as targeting CXCR2-mediated recruitment, CXCR4/CXCL12 niche retention, PAD4-dependent NET formation, neutrophil elastase activity, ARG1/ROS-mediated suppression, or DKK1-driven immature neutrophil states, may offer better therapeutic precision [94,122].

Finally, the field needs stronger standards for causal inference. Demonstrating that a mechanism is truly neutrophil-driven in bone metastasis should require more than increased neutrophil abundance or nonspecific myeloid depletion. Stronger evidence should combine spatial localization, functional assays, selective genetic or pharmacologic perturbation, rescue or adoptive-transfer experiments, and careful exclusion of indirect effects on monocytes, osteoclast-lineage cells, stromal cells, or hematopoietic progenitors [141,214]. These approaches will be essential for distinguishing causal neutrophil programs from bystander inflammation within the metastatic bone niche.

## 12. Conclusions

Neutrophil-lineage cells are emerging as important regulators of bone metastasis because they are produced, retained, mobilized, and reprogrammed within the same marrow ecosystem that disseminated tumor cells exploit for skeletal colonization. Current evidence links these cells to tumor-cell trafficking, marrow vascular arrest, immune suppression, NET-mediated matrix remodeling, dormancy escape, and modulation of osteoclast–osteoblast coupling. However, their effects are highly context-dependent and cannot be reduced to a single pro-tumor or anti-tumor identity.

The strongest bone-specific evidence comes from breast and prostate cancer models. In breast cancer, CXCR4/CXCL12-associated homing, CXCL5/CXCR2-driven colonization, and DKK1-mediated expansion of CHI3L3-positive immature suppressive neutrophils connect neutrophil-lineage cells to skeletal progression and impaired immunotherapy response. In prostate cancer, neutrophils appear to have stage-dependent roles, including early cytotoxic activity and later pro-metastatic support through mechanisms such as PR3–RAGE signaling and therapy-associated immune reprogramming.

Several mechanisms, including CTC–neutrophil clustering and NET-mediated dormancy awakening, are still supported mainly by non-skeletal metastatic models and require direct validation in bone. Future studies should combine spatial profiling, granulocyte-sensitive single-cell approaches, functional immune assays, and selective perturbation strategies to distinguish causal neutrophil programs from bystander inflammation. Therapeutically, the most promising direction is not indiscriminate neutrophil depletion, but selective inhibition or reprogramming of pathogenic neutrophil states while preserving host defense and potential anti-tumor neutrophil functions.

## Figures and Tables

**Table 1:** Representative clinical trials and clinical-stage strategies targeting neutrophil-lineage pathways, PMN-MDSCs, or NET-associated biology in solid tumors.

Strategy/Agent	Main Target	Clinical Setting	Trial/Source	Relevance to Skeletal Metastasis	Related Manuscript References
SX-682 + pembrolizumab	CXCR1/2 blockade; reduced suppressive myeloid recruitment	Metastatic melanoma	Phase 1; NCT03161431	Supports CXCR1/2 blockade with PD-1 inhibition; not bone-specific.	[72,143,178,203]
SX-682 + nivolumab	CXCR1/2 blockade plus PD-1 inhibition	Metastatic pancreatic ductal adenocarcinoma	Phase 1; NCT04477343	Tests CXCR1/2 inhibition in a myeloid-rich solid tumor; not bone-specific.	[143,178,203]
AZD5069 + enzalutamide	CXCR2 blockade plus androgen receptor inhibition	Metastatic castration-resistant prostate cancer	Phase 1/2; NCT03177187	Highly relevant because mCRPC is often bone-dominant.	[95,106,178,203]
Navarixin + pembrolizumab	CXCR2 blockade plus PD-1 inhibition	Advanced/metastatic CRPC, MSS colorectal cancer, and PD-L1+ NSCLC	Phase 2; NCT03473925	Useful cautionary example: feasible safety but limited efficacy.	[143,178,203,204]
Reparixin + paclitaxel	CXCR1/2 pathway inhibition	HER2-negative metastatic breast cancer	Phase Ib; NCT02001974	Provides breast cancer precedent for CXCR1/2 targeting; not bone-specific.	[72,106,178]
Tasquinimod	S100A9-associated myeloid/TME modulation	Metastatic castration-resistant prostate cancer	Phase 3; NCT01234311	Bone-relevant mCRPC example; improved rPFS but not OS.	[78,140,203]
Entinostat + pembrolizumab	HDAC inhibition; indirect MDSC modulation	Advanced solid tumors	Phase 1; NCT02909452	Example of indirect myeloid reprogramming with checkpoint blockade.	[132,140,200,201]
ATRA + cemiplimab	Myeloid differentiation/MDSC modulation	Advanced or metastatic leiomyosarcoma	Phase 2; NCT06528769	Illustrates differentiation-based myeloid reprogramming; not bone-specific.	[132,140,200,201]
DNase-based or PAD4-targeted NET approaches	NET degradation or inhibition of NET formation	No established skeletal-metastasis-specific trial identified	Clinical gap	Strong preclinical rationale, but no direct clinical validation in skeletal metastasis.	[97,119,122,127,202]

## Data Availability

No new data were created or analyzed in this study. Data sharing is not applicable to this article.

## References

[1] Hiraga T. (2025). Immune microenvironment of cancer bone metastasis. Bone.

[2] He N., Jiang J. (2022). Contribution of immune cells to bone metastasis pathogenesis. Front. Endocrinol..

[3] Zhang W., Bado I., Wang H., Lo H.-C., Zhang X.H.F. (2019). Bone Metastasis: Find Your Niche and Fit in. Trends Cancer.

[4] Haider M.T., Smit D.J., Taipaleenmäki H. (2020). The Endosteal Niche in Breast Cancer Bone Metastasis. Front. Oncol..

[5] Esposito M., Mondal N., Greco T.M., Wei Y., Spadazzi C., Lin S.-C., Zheng H., Cheung C., Magnani J.L., Lin S.-H. (2019). Bone vascular niche E-selectin induces mesenchymal–epithelial transition and Wnt activation in cancer cells to promote bone metastasis. Nat. Cell Biol..

[6] Price T.T., Burness M.L., Sivan A., Warner M.J., Cheng R., Lee C.H., Olivere L., Comatas K., Magnani J., Kim Lyerly H. (2016). Dormant breast cancer micrometastases reside in specific bone marrow niches that regulate their transit to and from bone. Sci. Transl. Med..

[7] Shupp A.B., Kolb A.D., Mukhopadhyay D., Bussard K.M. (2018). Cancer Metastases to Bone: Concepts, Mechanisms, and Interactions with Bone Osteoblasts. Cancers.

[8] Ottewell P.D. (2016). The role of osteoblasts in bone metastasis. J. Bone Oncol..

[9] Battafarano G., Rossi M., Marampon F., Del Fattore A. (2018). Cellular and Molecular Mediators of Bone Metastatic Lesions. Int. J. Mol. Sci..

[10] Clézardin P., Coleman R., Puppo M., Ottewell P., Bonnelye E., Paycha F., Confavreux C.B., Holen I. (2021). Bone metastasis: Mechanisms, therapies, and biomarkers. Physiol. Rev..

[11] Cheng X., Wang Z. (2021). Immune Modulation of Metastatic Niche Formation in the Bone. Front. Immunol..

[12] Granata V., Crisafulli L., Nastasi C., Ficara F., Sobacchi C. (2022). Bone Marrow Niches and Tumour Cells: Lights and Shadows of a Mutual Relationship. Front. Immunol..

[13] Jaillon S., Ponzetta A., Di Mitri D., Santoni A., Bonecchi R., Mantovani A. (2020). Neutrophil diversity and plasticity in tumour progression and therapy. Nat. Rev. Cancer.

[14] Xiang L., Gilkes D.M. (2019). The Contribution of the Immune System in Bone Metastasis Pathogenesis. Int. J. Mol. Sci..

[15] Dutta A., Bhagat S., Paul S., Katz J.P., Sengupta D., Bhargava D. (2023). Neutrophils in Cancer and Potential Therapeutic Strategies Using Neutrophil-Derived Exosomes. Vaccines.

[16] Giese M.A., Hind L.E., Huttenlocher A. (2019). Neutrophil plasticity in the tumor microenvironment. Blood.

[17] Liu Y., Sun Q., Guo J., Yan L., Yan Y., Gong Y., Lin J., Yuan H., Jin J., Wang B. (2025). Dual ferroptosis induction in N2-TANs and TNBC cells via FTH1 targeting: A therapeutic strategy for triple-negative breast cancer. Cell Rep. Med..

[18] Yan S., Zhao W., Du J., Teng L., Yu T., Xu P., Liu J., Yang R., Dong Y., Wang H. (2025). C-FOS promotes the formation of neutrophil extracellular traps and the recruitment of neutrophils in lung metastasis of triple-negative breast cancer. J. Exp. Clin. Cancer Res..

[19] Furumaya C., Martinez-Sanz P., Bouti P., Kuijpers T.W., Matlung H.L. (2020). Plasticity in Pro- and Anti-tumor Activity of Neutrophils: Shifting the Balance. Front. Immunol..

[20] Li P., Fan F., Zhang B., Yuan C., Liang H. (2025). Neutrophil Spatiotemporal Regulatory Networks: Dual Roles in Tumor Growth Regulation and Metastasis. Biomedicines.

[21] Kwantwi L.B. (2023). Interplay between tumor-derived factors and tumor-associated neutrophils: Opportunities for therapeutic interventions in cancer. Clin. Transl. Oncol..

[22] Gong Z., Li Q., Shi J., Li P., Hua L., Shultz L.D., Ren G. (2023). Immunosuppressive reprogramming of neutrophils by lung mesenchymal cells promotes breast cancer metastasis. Sci. Immunol..

[23] Zhou Y., Shen G., Zhou X., Li J. (2025). Therapeutic potential of tumor-associated neutrophils: Dual role and phenotypic plasticity. Signal Transduct. Target. Ther..

[24] Kaltenmeier C., Simmons R.L., Tohme S., Yazdani H.O. (2021). Neutrophil Extracellular Traps (NETs) in Cancer Metastasis. Cancers.

[25] Smith J.T., Chai R.C. (2024). Bone niches in the regulation of tumour cell dormancy. J. Bone Oncol..

[26] Nowosad A., Marine J.-C., Karras P. (2023). Perivascular niches: Critical hubs in cancer evolution. Trends Cancer.

[27] Busch C., Nyamondo K., Wheadon H. (2024). Complexities of modeling the bone marrow microenvironment to facilitate hematopoietic research. Exp. Hematol..

[28] Kim M.J., Valderrábano R.J., Wu J.Y. (2022). Osteoblast Lineage Support of Hematopoiesis in Health and Disease. J. Bone Miner. Res..

[29] Udagawa N., Koide M., Nakamura M., Nakamichi Y., Yamashita T., Uehara S., Kobayashi Y., Furuya Y., Yasuda H., Fukuda C. (2021). Osteoclast differentiation by RANKL and OPG signaling pathways. J. Bone Miner. Metab..

[30] Elson A., Anuj A., Barnea-Zohar M., Reuven N. (2022). The origins and formation of bone-resorbing osteoclasts. Bone.

[31] Chiba-Ohkuma R., Karakida T., Yamamoto R., Yamakoshi Y. (2025). Direct and indirect effects of transforming growth factor-beta on osteoclast-mediated bone remodeling using a new in vitro bone matrix model. JBMR Plus.

[32] Sugiyama T., Omatsu Y., Nagasawa T. (2019). Niches for hematopoietic stem cells and immune cell progenitors. Int. Immunol..

[33] Omatsu Y., Nagasawa T. (2021). Identification of microenvironmental niches for hematopoietic stem cells and lymphoid progenitors—Bone marrow fibroblastic reticular cells with salient features. Int. Immunol..

[34] Asada N., Takeishi S., Frenette P.S. (2017). Complexity of bone marrow hematopoietic stem cell niche. Int. J. Hematol..

[35] Yang P., Hu Y., Zhou Q. (2020). The CXCL12-CXCR4 Signaling Axis Plays a Key Role in Cancer Metastasis and is a Potential Target for Developing Novel Therapeutics against Metastatic Cancer. Curr. Med. Chem..

[36] Asada N., Kunisaki Y., Pierce H., Wang Z., Fernandez N.F., Birbrair A., Ma’ayan A., Frenette P.S. (2017). Differential cytokine contributions of perivascular haematopoietic stem cell niches. Nat. Cell Biol..

[37] Nakatani T., Sugiyama T., Omatsu Y., Watanabe H., Kondoh G., Nagasawa T. (2023). Ebf3+ niche-derived CXCL12 is required for the localization and maintenance of hematopoietic stem cells. Nat. Commun..

[38] Li Y., Cao S., Gaculenko A., Zhan Y., Bozec A., Chen X. (2022). Distinct Metabolism of Bone Marrow Adipocytes and their Role in Bone Metastasis. Front. Endocrinol..

[39] Wilson A., Garmo L.C., Podgorski I. (2022). Interplay between fat cells and immune cells in bone: Impact on malignant progression and therapeutic response. Pharmacol. Ther..

[40] Wu M.-Y., Li C.-J., Yiang G.-T., Cheng Y.-L., Tsai A.P.-Y., Hou Y.-T., Ho Y.-C., Hou M.-F., Chu P.-Y. (2018). Molecular Regulation of Bone Metastasis Pathogenesis. Cell. Physiol. Biochem..

[41] Song C., Tong T., Dai B., Zhu Y., Chen E., Zhang M., Zhang W. (2024). Osteoimmunology in bone malignancies: A symphony with evil. J. Natl. Cancer Cent..

[42] De Meo M.L., Spicer J.D. (2021). The role of neutrophil extracellular traps in cancer progression and metastasis. Semin. Immunol..

[43] Cheng H., Cheng Y., Wang G., Wang D.-C., Liu X. (2025). From Inflammation to Bone Loss: The Multifaceted Role of Neutrophils in Osteoporosis. Int. J. Inflamm..

[44] Shu L.-Z., Zhang X.-L., Ding Y.-D., Lin H. (2024). From inflammation to bone formation: The intricate role of neutrophils in skeletal muscle injury and traumatic heterotopic ossification. Exp. Mol. Med..

[45] Buijs J.T., Stayrook K.R., Guise T.A. (2012). The role of TGF-β in bone metastasis: Novel therapeutic perspectives. BoneKEy Rep..

[46] Esposito M., Guise T., Kang Y. (2018). The Biology of Bone Metastasis. Cold Spring Harb. Perspect. Med..

[47] Litak J., Czyżewski W., Szymoniuk M., Sakwa L., Pasierb B., Litak J., Hoffman Z., Kamieniak P., Roliński J. (2022). Biological and Clinical Aspects of Metastatic Spinal Tumors. Cancers.

[48] Young S.A.E., Heller A.-D., Garske D.S., Rummler M., Qian V., Ellinghaus A., Duda G.N., Willie B.M., Grüneboom A., Cipitria A. (2024). From breast cancer cell homing to the onset of early bone metastasis: The role of bone (re)modeling in early lesion formation. Sci. Adv..

[49] Baldessari C., Pipitone S., Molinaro E., Cerma K., Fanelli M., Nasso C., Oltrecolli M., Pirola M., D’Agostino E., Pugliese G. (2023). Bone Metastases and Health in Prostate Cancer: From Pathophysiology to Clinical Implications. Cancers.

[50] Bailey S., Stadelmann M.A., Zysset P.K., Vashishth D., Alkalay R.N. (2022). Influence of Metastatic Bone Lesion Type and Tumor Origin on Human Vertebral Bone Architecture, Matrix Quality, and Mechanical Properties. J. Bone Miner. Res..

[51] Sims N.A., Martin T.J. (2020). Osteoclasts Provide Coupling Signals to Osteoblast Lineage Cells Through Multiple Mechanisms. Annu. Rev. Physiol..

[52] Lerner U.H., Kindstedt E., Lundberg P. (2019). The critical interplay between bone resorbing and bone forming cells. J. Clin. Periodontol..

[53] Carmona-Rivera C., Kaplan M.J., O’Neil L.J. (2024). Neutrophils in Inflammatory Bone Diseases. Curr. Osteoporos. Rep..

[54] Hajishengallis G., Moutsopoulos N.M., Hajishengallis E., Chavakis T. (2016). Immune and regulatory functions of neutrophils in inflammatory bone loss. Semin. Immunol..

[55] Ando Y., Tsukasaki M., Huynh N.C.-N., Zang S., Yan M., Muro R., Nakamura K., Komagamine M., Komatsu N., Okamoto K. (2024). The neutrophil–osteogenic cell axis promotes bone destruction in periodontitis. Int. J. Oral Sci..

[56] Hensel J., Thalmann G.N. (2016). Biology of Bone Metastases in Prostate Cancer. Urology.

[57] Hsu B.E., Shen Y., Siegel P.M. (2020). Neutrophils: Orchestrators of the Malignant Phenotype. Front. Immunol..

[58] Thind M.K., Uhlig H.H., Glogauer M., Palaniyar N., Bourdon C., Gwela A., Lancioni C.L., Berkley J.A., Bandsma R.H.J., Farooqui A. (2023). A metabolic perspective of the neutrophil life cycle: New avenues in immunometabolism. Front. Immunol..

[59] Cowland J.B., Borregaard N. (2016). Granulopoiesis and granules of human neutrophils. Immunol. Rev..

[60] Evrard M., Kwok I.W.H., Chong S.Z., Teng K.W.W., Becht E., Chen J., Sieow J.L., Penny H.L., Ching G.C., Devi S. (2018). Developmental Analysis of Bone Marrow Neutrophils Reveals Populations Specialized in Expansion, Trafficking, and Effector Functions. Immunity.

[61] Signoretto I., Calzetti F., Finotti G., Lonardi S., Balanzin C., Bianchetto-Aguilera F., Gasperini S., Gardiman E., Castellucci M., Russignan A. (2025). Uncovering two neutrophil-committed progenitors that immediately precede promyelocytes during human neutropoiesis. Cell. Mol. Immunol..

[62] Zlotnik A., Burkhardt A.M., Homey B. (2011). Homeostatic chemokine receptors and organ-specific metastasis. Nat. Rev. Immunol..

[63] Strydom N., Rankin S.M. (2013). Regulation of circulating neutrophil numbers under homeostasis and in disease. J. Innate Immun..

[64] Suratt B.T., Petty J.M., Young S.K., Malcolm K.C., Lieber J.G., Nick J.A., Gonzalo J.A., Henson P.M., Worthen G.S. (2004). Role of the CXCR4/SDF-1 chemokine axis in circulating neutrophil homeostasis. Blood.

[65] Numata M., Hirano A., Yamamoto Y., Yasuda M., Miura N., Sayama K., Shibata M.A., Asai T., Oku N., Miyoshi N. (2021). Metastasis of Breast Cancer Promoted by Circadian Rhythm Disruption due to Light/Dark Shift and its Prevention by Dietary Quercetin in Mice. J. Circadian Rhythm..

[66] He X.Y., Gao Y., Ng D., Michalopoulou E., George S., Adrover J.M., Sun L., Albrengues J., Daßler-Plenker J., Han X. (2024). Chronic stress increases metastasis via neutrophil-mediated changes to the microenvironment. Cancer Cell.

[67] Shaul M.E., Fridlender Z.G. (2019). Tumour-associated neutrophils in patients with cancer. Nat. Rev. Clin. Oncol..

[68] Xiong S., Dong L., Cheng L. (2021). Neutrophils in cancer carcinogenesis and metastasis. J. Hematol. Oncol..

[69] Que H., Fu Q., Lan T., Tian X., Wei X. (2022). Tumor-associated neutrophils and neutrophil-targeted cancer therapies. Biochim. Biophys. Acta Rev. Cancer.

[70] Xu W., Zhu G., Sheng Y., Zhang W., Wang S., Wu Q. (2025). Tumor-Associated Neutrophils Regulate Breast Cancer Progression Through the AQP9/STAT3 Signaling Pathway. Cancer Sci..

[71] Lattanzi C., Bianchetto-Aguilera F., Donini M., Pettinella F., Caveggion E., Castellucci M., Gasperini S., Mariotti B., Signoretto I., Cantini M. (2025). Uncovering common transcriptional features shared by mature peripheral blood PMN-MDSCs and tumor-infiltrating neutrophils in humans. Oncoimmunology.

[72] Zhang R., Dong M., Tu J., Li F., Deng Q., Xu J., He X., Ding J., Xia J., Sheng D. (2023). PMN-MDSCs modulated by CCL20 from cancer cells promoted breast cancer cell stemness through CXCL2-CXCR2 pathway. Signal Transduct. Target. Ther..

[73] Zhang Y., Hu J., Zhang X., Liang M., Wang X., Gan D., Li J., Lu X., Wan J., Feng S. (2024). Protein Signature Differentiating Neutrophils and Myeloid-Derived Suppressor Cells Determined Using a Human Isogenic Cell Line Model and Protein Profiling. Cells.

[74] Hamam H.J., Palaniyar N. (2019). Post-Translational Modifications in NETosis and NETs-Mediated Diseases. Biomolecules.

[75] Korba-Mikołajczyk A., Służalska K.D., Kasperkiewicz P. (2025). Exploring the involvement of serine proteases in neutrophil extracellular traps: A review of mechanisms and implications. Cell Death Dis..

[76] Korkmaz B., Moreau T., Gauthier F. (2008). Neutrophil elastase, proteinase 3 and cathepsin G: Physicochemical properties, activity and physiopathological functions. Biochimie.

[77] Liu Y., Ma J., Ma Y., Wang B.Z., Wang Y., Yuan J., Zhang F., Zhao X., Chen K., Zhang X. (2025). Neutrophil extracellular traps impede cancer metastatic seeding via protease-activated receptor 2-mediated downregulation of phagocytic checkpoint CD24. J. Immunother. Cancer.

[78] Roca H., McCauley L.K. (2015). Inflammation and skeletal metastasis. BoneKEy Rep..

[79] Sato T., Takahashi S., Mizumoto T., Harao M., Akizuki M., Takasugi M., Fukutomi T., Yamashita J. (2006). Neutrophil elastase and cancer. Surg. Oncol..

[80] Guo J., Wang S., Gao Q. (2023). An integrated overview of the immunosuppression features in the tumor microenvironment of pancreatic cancer. Front. Immunol..

[81] Coffelt S.B., Wellenstein M.D., de Visser K.E. (2016). Neutrophils in cancer: Neutral no more. Nat. Rev. Cancer.

[82] Kowanetz M., Wu X., Lee J., Tan M., Hagenbeek T., Qu X., Yu L., Ross J., Korsisaari N., Cao T. (2010). Granulocyte-colony stimulating factor promotes lung metastasis through mobilization of Ly6G+Ly6C+ granulocytes. Proc. Natl. Acad. Sci. USA.

[83] Eash K.J., Greenbaum A.M., Gopalan P.K., Link D.C. (2010). CXCR2 and CXCR4 antagonistically regulate neutrophil trafficking from murine bone marrow. J. Clin. Investig..

[84] Goel P., Aryal S., Franceski A.M., Kuznetsova V., Costa A., Luca F., Connelly A.N., Phillips D.W., Ennis C.C., Curtiss B.M. (2025). The acute myeloid leukemia microenvironment impairs neutrophil maturation and function through NF-κB signaling. Blood.

[85] Tulotta C., Stefanescu C., Chen Q., Torraca V., Meijer A.H., Snaar-Jagalska B.E. (2019). CXCR4 signaling regulates metastatic onset by controlling neutrophil motility and response to malignant cells. Sci. Rep..

[86] Jablonska J., Lang S., Sionov R.V., Granot Z. (2017). The regulation of pre-metastatic niche formation by neutrophils. Oncotarget.

[87] Kaan Aydın V., Adalı Y., Köseler A. (2026). A comparative analysis of CXCR4 gene expression from published datasets and cell surface protein levels in breast and prostate cancer models. Biochem. Biophys. Res. Commun..

[88] Cordier C., Haustrate A., Mihalache A., Duval E., Desruelles E., Spriet C., Casel B., Slimani L., Soret B., Allart L. (2025). Targeting TRPV6/CXCR4 complexes prevents castration-resistant prostate cancer metastasis to the bone. Signal Transduct. Target. Ther..

[89] Kottmann V., Nienhaus M., Drees P., Gercek E., Ritz U. (2026). From bone homeostasis to skeletal metastasis and osteosarcoma: Insights into osteoclast and osteoblast roles in bone remodelling and cancer. Biochim. Biophys. Acta Rev. Cancer.

[90] Jia J., Wang Y., Li M., Wang F., Peng Y., Hu J., Li Z., Bian Z., Yang S. (2024). Neutrophils in the premetastatic niche: Key functions and therapeutic directions. Mol. Cancer.

[91] Chen E., Yu J. (2023). The role and metabolic adaptations of neutrophils in premetastatic niches. Biomark. Res..

[92] Wu L., Saxena S., Singh R.K. (2020). Neutrophils in the Tumor Microenvironment. Adv. Exp. Med. Biol..

[93] Sang L., Zhou X., Wu J. (2026). Review of the premetastatic niche in liver cancer bone metastases. Int. J. Clin. Exp. Pathol..

[94] Shi T., Liu W., Luo Y., Liang K., Ren S., Song X., Liu F., Lu C., Hirschhorn D., Wang H. (2025). CHI3L3(+) immature neutrophils inhibit anti-tumor immunity and impede immune checkpoint blockade therapy in bone metastases. Cancer Cell.

[95] Costanzo-Garvey D.L., Keeley T., Case A.J., Watson G.F., Alsamraae M., Yu Y., Su K., Heim C.E., Kielian T., Morrissey C. (2020). Neutrophils are mediators of metastatic prostate cancer progression in bone. Cancer Immunol. Immunother..

[96] Prakash J., Shaked Y. (2024). The Interplay between Extracellular Matrix Remodeling and Cancer Therapeutics. Cancer Discov..

[97] Wang Y., Yang K., Li J., Wang C., Li P., Du L. (2025). Neutrophil extracellular traps in cancer: From mechanisms to treatments. Clin. Transl. Med..

[98] Retter A., Singer M., Annane D. (2025). “The NET effect”: Neutrophil extracellular traps-a potential key component of the dysregulated host immune response in sepsis. Crit. Care.

[99] Xin Z., Qin L., Tang Y., Guo S., Li F., Fang Y., Li G., Yao Y., Zheng B., Zhang B. (2024). Immune mediated support of metastasis: Implication for bone invasion. Cancer Commun..

[100] Lin Q., Fang X., Liang G., Luo Q., Cen Y., Shi Y., Jia S., Li J., Yang W., Sanders A.J. (2021). Silencing CTNND1 Mediates Triple-Negative Breast Cancer Bone Metastasis via Upregulating CXCR4/CXCL12 Axis and Neutrophils Infiltration in Bone. Cancers.

[101] Su Y., Leng M., Yang Q., Jiang W., Xiang G., Long L., Zhou X. (2025). Targeting circulating tumor cell–neutrophil interactions: Nanoengineered strategies for inhibiting cancer metastasis. J. Nanobiotechnol..

[102] Schuster E., Taftaf R., Reduzzi C., Albert M.K., Romero-Calvo I., Liu H. (2021). Better together: Circulating tumor cell clustering in metastatic cancer. Trends Cancer.

[103] Sun R., Xiong Y., Liu H., Gao C., Su L., Weng J., Yuan X., Zhang D., Feng J. (2020). Tumor-associated neutrophils suppress antitumor immunity of NK cells through the PD-L1/PD-1 axis. Transl. Oncol..

[104] Hu C., Long L., Lou J., Leng M., Yang Q., Xu X., Zhou X. (2024). CTC-neutrophil interaction: A key driver and therapeutic target of cancer metastasis. Biomed. Pharmacother..

[105] Choi S.W., Sun A.K., Cheung J.P., Ho J.C. (2024). Circulating Tumour Cells in the Prediction of Bone Metastasis. Cancers.

[106] Romero-Moreno R., Curtis K.J., Coughlin T.R., Miranda-Vergara M.C., Dutta S., Natarajan A., Facchine B.A., Jackson K.M., Nystrom L., Li J. (2019). The CXCL5/CXCR2 axis is sufficient to promote breast cancer colonization during bone metastasis. Nat. Commun..

[107] Wu M., Ma M., Tan Z., Zheng H., Liu X. (2020). Neutrophil: A New Player in Metastatic Cancers. Front. Immunol..

[108] Di Russo S., Liberati F.R., Riva A., Di Fonzo F., Macone A., Giardina G., Arese M., Rinaldo S., Cutruzzolà F., Paone A. (2024). Beyond the barrier: The immune-inspired pathways of tumor extravasation. Cell Commun. Signal..

[109] Lin D., Shen L., Luo M., Zhang K., Li J., Yang Q., Zhu F., Zhou D., Zheng S., Chen Y. (2021). Circulating tumor cells: Biology and clinical significance. Signal Transduct. Target. Ther..

[110] Mahmud Z., Rahman A., Mishu I.D., Kabir Y. (2022). Mechanistic insights into the interplays between neutrophils and other immune cells in cancer development and progression. Cancer Metastasis Rev..

[111] Crippa M., Talò G., Lamouline A., Bolis S., Arrigoni C., Bersini S., Moretti M. (2022). A microfluidic model of human vascularized breast cancer metastasis to bone for the study of neutrophil-cancer cell interactions. Mater. Today Bio..

[112] Sionov R.V. (2021). Leveling Up the Controversial Role of Neutrophils in Cancer: When the Complexity Becomes Entangled. Cells.

[113] Li X., Wang M., Gong T., Lei X., Hu T., Tian M., Ding F., Ma F., Chen H., Liu Z. (2020). A S100A14-CCL2/CXCL5 signaling axis drives breast cancer metastasis. Theranostics.

[114] Hu W., Zhang L., Dong Y., Tian Z., Chen Y., Dong S. (2020). Tumour dormancy in inflammatory microenvironment: A promising therapeutic strategy for cancer-related bone metastasis. Cell Mol. Life Sci..

[115] Yu-Lee L.Y., Yu G., Lee Y.C., Lin S.C., Pan J., Pan T., Yu K.J., Liu B., Creighton C.J., Rodriguez-Canales J. (2018). Osteoblast-Secreted Factors Mediate Dormancy of Metastatic Prostate Cancer in the Bone via Activation of the TGFβRIII-p38MAPK-pS249/T252RB Pathway. Cancer Res..

[116] Sosa M.S., Parikh F., Maia A.G., Estrada Y., Bosch A., Bragado P., Ekpin E., George A., Zheng Y., Lam H.-M. (2015). NR2F1 controls tumour cell dormancy via SOX9- and RARβ-driven quiescence programmes. Nat. Commun..

[117] Khalil B.D., Sanchez R., Rahman T., Rodriguez-Tirado C., Moritsch S., Martinez A.R., Miles B., Farias E., Mezei M., Nobre A.R. (2021). An NR2F1-specific agonist suppresses metastasis by inducing cancer cell dormancy. J. Exp. Med..

[118] Widner D.B., Park S.H., Eber M.R., Shiozawa Y. (2018). Interactions Between Disseminated Tumor Cells and Bone Marrow Stromal Cells Regulate Tumor Dormancy. Curr. Osteoporos. Rep..

[119] Adrover J.M., McDowell S.A.C., He X.Y., Quail D.F., Egeblad M. (2023). NETworking with cancer: The bidirectional interplay between cancer and neutrophil extracellular traps. Cancer Cell.

[120] Mei S., Alchahin A.M., Embaie B.T., Gavriliuc I.M., Verhoeven B.M., Zhao T., Li X., Jeffries N.E., Pepich A., Sarkar H. (2024). Single-cell analyses of metastatic bone marrow in human neuroblastoma reveals microenvironmental remodeling and metastatic signature. J. Clin. Investig..

[121] Albrengues J., Shields M.A., Ng D., Park C.G., Ambrico A., Poindexter M.E., Upadhyay P., Uyeminami D.L., Pommier A., Küttner V. (2018). Neutrophil extracellular traps produced during inflammation awaken dormant cancer cells in mice. Science.

[122] Deng H., Lin C., Garcia-Gerique L., Fu S., Cruz Z., Bonner E.E., Rosenwasser M., Rajagopal S., Sadhu M.N., Gajendran C. (2022). A Novel Selective Inhibitor JBI-589 Targets PAD4-Mediated Neutrophil Migration to Suppress Tumor Progression. Cancer Res..

[123] Isojima T., Crimeen-Irwin B., McGregor N.E., Chai R.C., Poulton I.J., Walker E.C., Dutt M., Parker B.L., Sims N.A. (2025). Bone marrow neutrophil progenitors suppress osteoclast formation in murine cortical and trabecular bone. Blood.

[124] Norouzi F., Eini P., Tahmasebi S. (2025). The role of NETosis in breast cancer: Mechanistic insights and biomarker potential. Breast Cancer Res..

[125] Liu Y., Guo F., Han Z., Yin Y., Chen G., Zhang Y., Tang Q., Chen L. (2025). Neutrophils inhibit bone formation by directly contacting osteoblasts and suppressing osteogenic differentiation. Bone.

[126] Yang L., Liu Q., Zhang X., Liu X., Zhou B., Chen J., Huang D., Li J., Li H., Chen F. (2020). DNA of neutrophil extracellular traps promotes cancer metastasis via CCDC25. Nature.

[127] Zhu W., Yang S., Meng D., Wang Q., Ji J. (2023). Targeting NADPH Oxidase and Integrin α5β1 to Inhibit Neutrophil Extracellular Traps-Mediated Metastasis in Colorectal Cancer. Int. J. Mol. Sci..

[128] Genna A., Vanwynsberghe A.M., Villard A.V., Pottier C., Ancel J., Polette M., Gilles C. (2020). EMT-Associated Heterogeneity in Circulating Tumor Cells: Sticky Friends on the Road to Metastasis. Cancers.

[129] Martins-Cardoso K., Maçao A., Souza J.L., Silva A.G., König S., Martins-Gonçalves R., Hottz E.D., Rondon A.M.R., Versteeg H.H., Bozza P.T. (2023). TF/PAR2 Signaling Axis Supports the Protumor Effect of Neutrophil Extracellular Traps (NETs) on Human Breast Cancer Cells. Cancers.

[130] Xia X., Zhang Z., Zhu C., Ni B., Wang S., Yang S., Yu F., Zhao E., Li Q., Zhao G. (2022). Neutrophil extracellular traps promote metastasis in gastric cancer patients with postoperative abdominal infectious complications. Nat. Commun..

[131] He D., Wu Q., Tian P., Liu Y., Jia Z., Li Z., Wang Y., Jin Y., Luo W., Li L. (2025). Chemotherapy awakens dormant cancer cells in lung by inducing neutrophil extracellular traps. Cancer Cell.

[132] Raskov H., Orhan A., Gaggar S., Gögenur I. (2022). Neutrophils and polymorphonuclear myeloid-derived suppressor cells: An emerging battleground in cancer therapy. Oncogenesis.

[133] Ramachandran I.R., Condamine T., Lin C., Herlihy S.E., Garfall A., Vogl D.T., Gabrilovich D.I., Nefedova Y. (2016). Bone marrow PMN-MDSCs and neutrophils are functionally similar in protection of multiple myeloma from chemotherapy. Cancer Lett..

[134] Patel S., Fu S., Mastio J., Dominguez G.A., Purohit A., Kossenkov A., Lin C., Alicea-Torres K., Sehgal M., Nefedova Y. (2018). Unique pattern of neutrophil migration and function during tumor progression. Nat. Immunol..

[135] Wen J., Huang G., Liu S., Wan J., Wang X., Zhu Y., Kaliney W., Zhang C., Cheng L., Wen X. (2020). Polymorphonuclear MDSCs are enriched in the stroma and expanded in metastases of prostate cancer. J. Pathol. Clin. Res..

[136] Antuamwine B.B., Bosnjakovic R., Hofmann-Vega F., Wang X., Theodosiou T., Iliopoulos I., Brandau S. (2023). N1 versus N2 and PMN-MDSC: A critical appraisal of current concepts on tumor-associated neutrophils and new directions for human oncology. Immunol. Rev..

[137] Li C., Xue Y., Yinwang E., Ye Z. (2025). The Recruitment and Immune Suppression Mechanisms of Myeloid-Derived Suppressor Cells and Their Impact on Bone Metastatic Cancer. Cancer Rep..

[138] Gabrilovich D.I., Nagaraj S. (2009). Myeloid-derived suppressor cells as regulators of the immune system. Nat. Rev. Immunol..

[139] Danilin S., Merkel A.R., Johnson J.R., Johnson R.W., Edwards J.R., Sterling J.A. (2012). Myeloid-derived suppressor cells expand during breast cancer progression and promote tumor-induced bone destruction. Oncoimmunology.

[140] Kusmartsev S. (2025). Metastasis-promoting functions of myeloid cells. Cancer Metastasis Rev..

[141] Capietto A.-H., Lee S., Clever D., Eul E., Ellis H., Ma C.X., Faccio R. (2021). Effective Treatment of Established Bone Metastases Can Be Achieved by Combinatorial Osteoclast Blockade and Depletion of Granulocytic Subsets. Cancer Immunol. Res..

[142] Masucci M.T., Minopoli M., Carriero M.V. (2019). Tumor Associated Neutrophils. Their Role in Tumorigenesis, Metastasis, Prognosis and Therapy. Front. Oncol..

[143] Sun L., Clavijo P.E., Robbins Y., Patel P., Friedman J., Greene S., Das R., Silvin C., Van Waes C., Horn L.A. (2019). Inhibiting myeloid-derived suppressor cell trafficking enhances T cell immunotherapy. J. Clin. Investig..

[144] Haider M.-T., Ridlmaier N., Smit D.J., Taipaleenmäki H. (2021). Interleukins as Mediators of the Tumor Cell—Bone Cell Crosstalk during the Initiation of Breast Cancer Bone Metastasis. Int. J. Mol. Sci..

[145] Maroni P., Bendinelli P., Ferraretto A., Lombardi G. (2021). Interleukin 11 (IL-11): Role(s) in Breast Cancer Bone Metastases. Biomedicines.

[146] Liu L., Liu Y., Yan X., Zhou C., Xiong X. (2020). The role of granulocyte colony-stimulating factor in breast cancer development: A review. Mol. Med. Rep..

[147] Song X., Wei C., Li X. (2022). The Signaling Pathways Associated With Breast Cancer Bone Metastasis. Front. Oncol..

[148] Zhang Y., Liang J., Liu P., Wang Q., Liu L., Zhao H. (2022). The RANK/RANKL/OPG system and tumor bone metastasis: Potential mechanisms and therapeutic strategies. Front. Endocrinol..

[149] Akhund S.A., Said K., Mohammad K.S. (2023). Mechanism of TGFβ in Bone Metastases and its Potential Therapeutic Uses. J. Orthop. Res. Ther..

[150] Khosravi M., Rostami Faradonbeh N. (2025). The Multicellular Vicious Cycle of Bone Metastasis: Reciprocal Interactions Between Tumor Cells and the Bone Microenvironment. Cancer Rev..

[151] Trivedi T., Pagnotti G.M., Guise T.A., Mohammad K.S. (2021). The Role of TGF-β in Bone Metastases. Biomolecules.

[152] Zhang W., Wang H., Sun M., Deng X., Wu X., Ma Y., Li M., Shuoa S.M., You Q., Miao L. (2020). CXCL5/CXCR2 axis in tumor microenvironment as potential diagnostic biomarker and therapeutic target. Cancer Commun..

[153] Midavaine É., Côté J., Sarret P. (2021). The multifaceted roles of the chemokines CCL2 and CXCL12 in osteophilic metastatic cancers. Cancer Metastasis Rev..

[154] Cambier S., Gouwy M., Proost P. (2023). The chemokines CXCL8 and CXCL12: Molecular and functional properties, role in disease and efforts towards pharmacological intervention. Cell. Mol. Immunol..

[155] Vičić I., Belev B. (2021). The pathogenesis of bone metastasis in solid tumors: A review. Croat. Med. J..

[156] Kfoury Y., Baryawno N., Severe N., Mei S., Gustafsson K., Hirz T., Brouse T., Scadden E.W., Igolkina A.A., Kokkaliaris K. (2021). Human prostate cancer bone metastases have an actionable immunosuppressive microenvironment. Cancer Cell.

[157] Alsamraae M., Costanzo-Garvey D., Teply B.A., Boyle S., Sommerville G., Herbert Z.T., Morrissey C., Dafferner A.J., Abdalla M.Y., Fallet R.W. (2023). Androgen receptor inhibition suppresses anti-tumor neutrophil response against bone metastatic prostate cancer via regulation of TβRI expression. Cancer Lett..

[158] Liao T., Chen W., Sun J., Zhang Y., Hu X., Yang S., Qiu H., Li S., Chu T. (2018). CXCR4 Accelerates Osteoclastogenesis Induced by Non-Small Cell Lung Carcinoma Cells Through Self-Potentiation and VCAM1 Secretion. Cell. Physiol. Biochem..

[159] Taverna S., Pucci M., Giallombardo M., Di Bella M.A., Santarpia M., Reclusa P., Gil-Bazo I., Rolfo C., Alessandro R. (2017). Amphiregulin contained in NSCLC-exosomes induces osteoclast differentiation through the activation of EGFR pathway. Sci. Rep..

[160] Brunetti G., Belisario D.C., Bortolotti S., Storlino G., Colaianni G., Faienza M.F., Sanesi L., Alliod V., Buffoni L., Centini E. (2020). LIGHT/TNFSF14 Promotes Osteolytic Bone Metastases in Non-small Cell Lung Cancer Patients. J. Bone Miner. Res..

[161] Sheervalilou M., Ghanei M., Arabfard M. (2025). Tumor-associated neutrophils and neutrophil extracellular traps in lung cancer: Antitumor/protumor insights and therapeutic implications. Med. Oncol..

[162] Zhang S., Sun L., Zuo J., Feng D. (2024). Tumor associated neutrophils governs tumor progression through an IL-10/STAT3/PD-L1 feedback signaling loop in lung cancer. Transl. Oncol..

[163] Aloe C., Wang H., Vlahos R., Irving L., Steinfort D., Bozinovski S. (2021). Emerging and multifaceted role of neutrophils in lung cancer. Transl. Lung Cancer Res..

[164] Chen K.C., Huang Y.H., Hsu K.H., Tseng J.S., Chang G.C., Yang T.Y. (2023). The Role of Neutrophil-to-Lymphocyte Ratio in Advanced EGFR-Mutant NSCLC Patients Treated with First-Line Osimertinib. OncoTargets Ther..

[165] Thio Q., Goudriaan W.A., Janssen S.J., Paulino Pereira N.R., Sciubba D.M., Rosovksy R.P., Schwab J.H. (2018). Prognostic role of neutrophil-to-lymphocyte ratio and platelet-to-lymphocyte ratio in patients with bone metastases. Br. J. Cancer.

[166] Ren Z., Yang J., Liang J., Xu Y., Lu G., Han Y., Zhu J., Tan H., Xu T., Ren M. (2022). Monitoring of postoperative neutrophil-to-lymphocyte ratio, D-dimer, and CA153 in: Diagnostic value for recurrent and metastatic breast cancer. Front. Surg..

[167] García-Ortega D.Y., Melendez-Fernandez A.P., Alvarez-Cano A., Clara-Altamirano M.A., Caro-Sanchez C., Alamilla-Garcia G., Luna-Ortiz K. (2022). Neutrophil-to-Lymphocyte ratio as a prognostic biomarker in extremities undifferentiated pleomorphic sarcoma. Surg. Oncol..

[168] Kaltenmeier C., Yazdani H.O., Morder K., Geller D.A., Simmons R.L., Tohme S. (2021). Neutrophil Extracellular Traps Promote T Cell Exhaustion in the Tumor Microenvironment. Front. Immunol..

[169] Zhang M., Wang X., Liu L., Ni Y., Zhang Y., Hu X., Huang C., Gong B., She T., Chen C. (2025). Bone marrow aspirate Cit-H3 was identified as a novel biomarker of minimal residual disease in neuroblastoma. BMC Cancer.

[170] Ihle C.L., Straign D.M., Canari J.A., Torkko K.C., Zolman K.L., Smith E.E., Owens P. (2024). Unique macrophage phenotypes activated by BMP signaling in breast cancer bone metastases. J. Clin. Investig..

[171] Futosi K., Fodor S., Mócsai A. (2013). Neutrophil cell surface receptors and their intracellular signal transduction pathways. Int. Immunopharmacol..

[172] Lerman I., Hammes S.R. (2018). Neutrophil elastase in the tumor microenvironment. Steroids.

[173] Paulsen G., Egner I., Raastad T., Reinholt F., Owe S., Lauritzen F., Brorson S.H., Koskinen S. (2013). Inflammatory markers CD11b, CD16, CD66b, CD68, myeloperoxidase and neutrophil elastase in eccentric exercised human skeletal muscles. Histochem. Cell Biol..

[174] Jia W., Mao Y., Luo Q., Wu J., Guan Q. (2024). Targeting neutrophil elastase is a promising direction for future cancer treatment. Discov. Oncol..

[175] Ingersoll S.A., Laval J., Forrest O.A., Preininger M., Brown M.R., Arafat D., Gibson G., Tangpricha V., Tirouvanziam R. (2015). Mature cystic fibrosis airway neutrophils suppress T cell function: Evidence for a role of arginase 1 but not programmed death-ligand 1. J. Immunol..

[176] Tyrinova T.V., Batorov E.V., Aristova T.A., Ushakova G.Y., Sizikova S.A., Denisova V.V., Ostanin A.A., Chernykh E.R. (2022). Expression of Inhibitory Molecules (Arginase-1, IDO, and PD-L1) by Myeloid-Derived Suppressor Cells in Multiple Myeloma Patients in Remission. Bull. Exp. Biol. Med..

[177] Schyrr F., Alonso-Calleja A., Vijaykumar A., Sordet-Dessimoz J., Gebhard S., Sarkis R., Bataclan C., Ferreira Lopes S., Oggier A., de Leval L. (2024). Inducible CXCL12/CXCR4-dependent extramedullary hematopoietic niches in the adrenal gland. Blood.

[178] Lazennec G., Rajarathnam K., Richmond A. (2024). CXCR2 chemokine receptor—A master regulator in cancer and physiology. Trends Mol. Med..

[179] Huang H., Zhang H., Onuma A.E., Tsung A. (2020). Neutrophil Elastase and Neutrophil Extracellular Traps in the Tumor Microenvironment. Adv. Exp. Med. Biol..

[180] Huang S., Shi J., Shen J., Fan X. (2025). Metabolic reprogramming of neutrophils in the tumor microenvironment: Emerging therapeutic targets. Cancer Lett..

[181] Liu Y., He M., Tang H., Xie T., Lin Y., Liu S., Liang J., Li F., Luo K., Yang M. (2024). Single-cell and spatial transcriptomics reveal metastasis mechanism and microenvironment remodeling of lymph node in osteosarcoma. BMC Med..

[182] Wan G., Maliga Z., Yan B., Vallius T., Shi Y., Khattab S., Chang C., Nirmal A.J., Yu K.H., Liu D. (2023). SpatialCells: Automated Profiling of Tumor Microenvironments with Spatially Resolved Multiplexed Single-Cell Data. bioRxiv.

[183] Grieshaber-Bouyer R., Nigrovic P.A. (2019). Neutrophil Heterogeneity as Therapeutic Opportunity in Immune-Mediated Disease. Front. Immunol..

[184] Guo W., Luan J., Huang X., Leon D., Gang S., Nicholson B., Bertacchi B., Bolotin D., Lingen M.W., Pearson A.T. (2026). Tumor-initiating stem cells fine-tune the plasticity of neutrophils to sculpt a protective niche. Cancer Cell.

[185] Wang T., Chen Z., Wang W., Wang H., Li S. (2025). Single-cell and spatial transcriptomic analysis reveals tumor cell heterogeneity and underlying molecular program in colorectal cancer. Front. Immunol..

[186] Xu Z., Liu F., Ding Y., Pan T., Wu Y.H., Han Y., Liu J., Bado I.L., Zhang W., Wu L. (2026). Unbiased niche labeling maps immune-excluded niche in bone metastasis. Cell.

[187] Timaxian C., Vogel C.F.A., Orcel C., Vetter D., Durochat C., Chinal C., Nguyen P., Aknin M.L., Mercier-Nomé F., Davy M. (2021). Pivotal Role for Cxcr2 in Regulating Tumor-Associated Neutrophil in Breast Cancer. Cancers.

[188] Kwak J.W., Nguyen H.Q., Camai A., Huffman G.M., Mekvanich S., Kenney N.N., Zhu X., Randolph T.W., Houghton A.M. (2024). CXCR1/2 antagonism inhibits neutrophil function and not recruitment in cancer. Oncoimmunology.

[189] Scala S. (2015). Molecular Pathways: Targeting the CXCR4-CXCL12 Axis--Untapped Potential in the Tumor Microenvironment. Clin. Cancer Res..

[190] Trautmann F., Cojoc M., Kurth I., Melin N., Bouchez L.C., Dubrovska A., Peitzsch C. (2014). CXCR4 as biomarker for radioresistant cancer stem cells. Int. J. Radiat. Biol..

[191] Park B.H., Kook S., Lee S., Jeong J.H., Brufsky A., Lee B.C. (2013). An isoform of C/EBPβ, LIP, regulates expression of the chemokine receptor CXCR4 and modulates breast cancer cell migration. J. Biol. Chem..

[192] Robinson T., Escara-Wilke J., Dai J., Zimmermann J., Keller E.T. (2023). A CXCR4 inhibitor (balixafortide) enhances docetaxel-mediated antitumor activity in a murine model of prostate cancer bone metastasis. Prostate.

[193] Mardomi A., Sabzichi M., Hussein Somi M., Shanehbandi D., Rahbarghazi R., Taj Sanjarani O., Samadi N. (2016). Trafficking mechanism of bone marrow-derived mesenchymal stem cells toward hepatocellular carcinoma HepG2 cells by modulating Endoglin, CXCR4 and TGF-β. Cell. Mol. Biol..

[194] Miao M., De Clercq E., Li G. (2020). Clinical significance of chemokine receptor antagonists. Expert Opin. Drug Metab. Toxicol..

[195] Cheng Q., Khodadadi L., Taddeo A., Klotsche J., Hoyer B.F., Radbruch A., Hiepe F. (2018). CXCR4-CXCL12 interaction is important for plasma cell homing and survival in NZB/W mice. Eur. J. Immunol..

[196] Zeng J., Xu H., Fan P.Z., Xie J., He J., Yu J., Gu X., Zhang C.J. (2020). Kaempferol blocks neutrophil extracellular traps formation and reduces tumour metastasis by inhibiting ROS-PAD4 pathway. J. Cell. Mol. Med..

[197] Xu J., Shi Q., Zeng F., Ren T., Wei R., Tang X. (2025). Neutrophil elastase promotes low molecular weight cyclin E1 formation to accelerate osteosarcoma proliferation. Front. Immunol..

[198] Qi L., Gao T., Bai C., Guo Z., Zhou L., Yang X., Fan Z., Zhang G. (2024). AOC3 accelerates lung metastasis of osteosarcoma by recruiting tumor-associated neutrophils, neutrophil extracellular trap formation and tumor vascularization. Heliyon.

[199] Islam M.M., Takeyama N. (2023). Role of Neutrophil Extracellular Traps in Health and Disease Pathophysiology: Recent Insights and Advances. Int. J. Mol. Sci..

[200] Zhang F., Xia Y., Su J., Quan F., Zhou H., Li Q., Feng Q., Lin C., Wang D., Jiang Z. (2024). Neutrophil diversity and function in health and disease. Signal Transduct. Target. Ther..

[201] Xu H., Chen X., Lu Y., Sun N., Weisgerber K.E., Xu M., Bai R.Y. (2025). Neutrophil Dynamics in Response to Cancer Therapies. Cancers.

[202] Zhang H., Wang Y., Onuma A., He J., Wang H., Xia Y., Lal R., Cheng X., Kasumova G., Hu Z. (2021). Neutrophils Extracellular Traps Inhibition Improves PD-1 Blockade Immunotherapy in Colorectal Cancer. Cancers.

[203] Hedrick C.C., Malanchi I. (2022). Neutrophils in cancer: Heterogeneous and multifaceted. Nat. Rev. Immunol..

[204] Ocana A., Nieto-Jiménez C., Pandiella A., Templeton A.J. (2017). Neutrophils in cancer: Prognostic role and therapeutic strategies. Mol. Cancer.

[205] Deng M., Ding H., Zhou Y., Qi G., Gan J. (2025). Cancer metastasis to the bone: Mechanisms and animal models (Review). Oncol. Lett..

[206] Jinnah A.H., Zacks B.C., Gwam C.U., Kerr B.A. (2018). Emerging and Established Models of Bone Metastasis. Cancers.

[207] Wakefield L., Agarwal S., Tanner K. (2023). Preclinical models for drug discovery for metastatic disease. Cell.

[208] Zheng Y., Sefik E., Astle J., Karatepe K., Öz H.H., Solis A.G., Jackson R., Luo H.R., Bruscia E.M., Halene S. (2022). Human neutrophil development and functionality are enabled in a humanized mouse model. Proc. Natl. Acad. Sci. USA.

[209] Nauseef W.M. (2023). Human neutrophils ≠ murine neutrophils: Does it matter?. Immunol. Rev..

[210] Cerezo-Wallis D., Rubio-Ponce A., Richter M., Pitino E., Kwok I., Marteletto G., Guanolema-Coba A.C., Shih C., Huang R.-K., Moraga A. (2026). Architecture of the neutrophil compartment. Nature.

[211] Klughammer J., Abravanel D.L., Segerstolpe Å., Blosser T.R., Goltsev Y., Cui Y., Goodwin D.R., Sinha A., Ashenberg O., Slyper M. (2024). A multi-modal single-cell and spatial expression map of metastatic breast cancer biopsies across clinicopathological features. Nat. Med..

[212] Li X., He X., Cao S., Shih C., Chen X., Ng L.G. (2026). Mapping of Neutrophils in Cancers: Insights From Spatial Omics Technologies. Eur. J. Immunol..

[213] Qin J., Wei F., Ren X. (2024). Neutrophils in the era of single-cell RNA sequencing: Functions and targeted therapies in cancer. Cancer Biol. Med..

[214] Boivin G., Faget J., Ancey P.-B., Gkasti A., Mussard J., Engblom C., Pfirschke C., Contat C., Pascual J., Vazquez J. (2020). Durable and controlled depletion of neutrophils in mice. Nat. Commun..

[215] Zhang J., Miao C., Zhang H. (2025). Targeting neutrophil extracellular traps in cancer progression and metastasis. Theranostics.

[216] Teijeira Á., Garasa S., Gato M., Alfaro C., Migueliz I., Cirella A., de Andrea C., Ochoa M.C., Otano I., Etxeberria I. (2020). CXCR1 and CXCR2 Chemokine Receptor Agonists Produced by Tumors Induce Neutrophil Extracellular Traps that Interfere with Immune Cytotoxicity. Immunity.

